# MRI Simulation Study Investigating Effects of Vessel Topology, Diffusion, and Susceptibility on Transverse Relaxation Rates Using a Cylinder Fork Model

**DOI:** 10.1038/s41598-017-15968-4

**Published:** 2017-11-24

**Authors:** Mohammed Salman Shazeeb, Jayashree Kalpathy-Cramer, Bashar Issa

**Affiliations:** 1 Department of Physics, College of Science, UAE University, Al-Ain, UAE; 2 Department of Radiology, Athinoula A. Martinos Center for Biomedical Imaging, Massachusetts General Hospital and Harvard Medical School, Boston, MA USA

## Abstract

Brain vasculature is conventionally represented as straight cylinders when simulating blood oxygenation level dependent (BOLD) contrast effects in functional magnetic resonance imaging (fMRI). In reality, the vasculature is more complicated with branching and coiling especially in tumors. Diffusion and susceptibility changes can also introduce variations in the relaxation mechanisms within tumors. This study introduces a simple cylinder fork model (CFM) and investigates the effects of vessel topology, diffusion, and susceptibility on the transverse relaxation rates R2* and R2. Simulations using Monte Carlo methods were performed to quantify R2* and R2 by manipulating the CFM at different orientations, bifurcation angles, and rotation angles. Other parameters of the CFM were chosen based on physiologically relevant values: vessel diameters (~2‒10 µm), diffusion rates (1 × 10^−11^‒1 × 10^−9^ m^2^/s), and susceptibility values (3 × 10^−8^–4 × 10^−7^ cgs units). R2* and R2 measurements showed a significant dependence on the bifurcation and rotation angles in several scenarios using different vessel diameters, orientations, diffusion rates, and susceptibility values. The angular dependence of R2* and R2 using the CFM could potentially be exploited as a tool to differentiate between normal and tumor vessels. The CFM can also serve as the elementary building block to simulate a capillary network reflecting realistic topological features.

## Introduction

Nearly three decades ago, Ogawa *et al*. discovered blood oxygenation level dependent (BOLD) contrast effects in functional magnetic resonance imaging (fMRI), which has come to dominate the field of brain mapping research^1^. Despite its widespread use as a tool in neuroscience, the physiological changes that underlie the BOLD effect have not been completely elucidated. In particular, the specific characteristics that affect the transverse relaxation rate changes (∆R2* and ∆R2), due to variation in blood volume and oxygenation in the brain vasculature occurring in active brain tissue, have not been fully characterized. Several studies have probed into this relationship by studying the effect of perturber geometry by assuming spheres or infinite cylinders oriented randomly to explore susceptibility contrast mechanisms^1–8^. Other studies have attempted to explore the use of microvasculature models to depict a more realistic *in vivo* scenario of the brain vasculature and its influence of vascular morphology effect on BOLD contrast^9–11^. In reality, however, the cortical vasculature is more complicated with meshwork geometry that contains a multitude of microscopic patterns and scales^12–19^.

The morphology and spatial arrangement of blood vessel networks in the brain exhibit certain design patterns that result in efficient delivery of oxygen and nutrients to the neural tissue. Some of these patterns have been described by Duvernoy *et al*. to include sinuous branches, glomerular loops, brush bristle and star-like appearances of vessel networks, and right angle penetration of vessels^12^. Motti *et al*. also described the stirrup, T-, and Y-morphology of vessels^13^. In tumorous tissue, the blood vessel network topology changes drastically, not only with increased vessel size and length, but also with increased tortuosity, sinusoidal branching, loops, bifurcations and trifurcations^13–19^. All these topographical traits can be generalized as possessing angular characteristics which become more distinct in tumorous tissue compared to normal tissue. Hence, exploring the idea that the shape and angular orientation of vessels can have an influence on the transverse relaxation rates (R2* and R2) could provide some useful insight.

The diffusion of protons due to random Brownian motion through magnetic field inhomogeneities is another factor that contributes to phase coherence changes and affects the transverse relaxation rates^4^. Thus, R2* and R2 will depend upon the diffusion coefficient of spins that are within the vicinity of the induced field inhomogeneities due to the presence of vessels. The extent of effect will also depend upon the correlation time (*τ*_*D*_) compared to the Larmor frequency variation (*∆ω*) at the vessel perturber surface^3^. For a vessel, these quantities are defined as:

1 $${\tau }_{D}={R}^{2}/D$$

2 $${\rm{\Delta }}\omega =\gamma \cdot {B}_{eq}(R)$$

where *R* is the radius of the vessel, *D* is the water diffusion coefficient (or diffusion rate), *γ* is the gyromagnetic ratio, and *B*_*eq*_ *(R)* is the equatorial magnetic field evaluated on the vessel surface. The values of the *τ*_*D*_ and *∆ω* will determine whether the diffusion rate falls in the motional averaged regime (MAR) or the static dephasing regime (SDR).

For a small vessel radius or equivalently fast diffusion rates, in the MAR (satisfying *∆ω*∙*τ*_*D*_ < 1), both R2* and R2 are equal and given by^20,21^:

3 $${(R{2}^{\ast })}^{MAR}={(R2)}^{SDR}=(\frac{16}{45})\cdot {({\rm{\Delta }}\omega )}^{2}\cdot f\cdot {\tau }_{D}$$

where *f* is the vessel volume fraction. For a large vessel size, relaxation is independent of the vessel radius and reaches an upper limit. In the SDR (satisfying *∆ω*∙*τ*_*D*_ > 1), R2* is given by^5,22^:

4 $${(R{2}^{\ast })}^{SDR}=(\frac{2\pi }{\sqrt{27}})\cdot {\rm{\Delta }}\omega \cdot f$$

As the radiofrequency (RF) pulse spacing (Carr-Purcell echo-time *τ*_*CP*_) is reduced for large vessels, the RF pulses become more effective and partial refocusing occurs causing R2 to decrease compared to R2*. In this regime, known as the echo-limited regime (ELR, valid for *τ*_*D*_ > 2∙*τ*_*CP*_), where *τ*_*CP*_ = TE/2, R2 is given by^23,24^:

5 $${(R2)}^{ELR}=\frac{7.2\,\cdot f\cdot {x}^{1/3}\cdot {(1.52+f\cdot x)}^{5/3}}{4\cdot {R}^{2}}$$

where *x* = *∆ω*∙*τ*_*CP*_. Since the state of water diffusion differs between normal and tumorous tissue^25^, studying the effect of diffusion, in combination with the vessel angular characteristics mentioned earlier, on R2* and R2 could further help characterize BOLD contrast effects.

**Figure 1:**
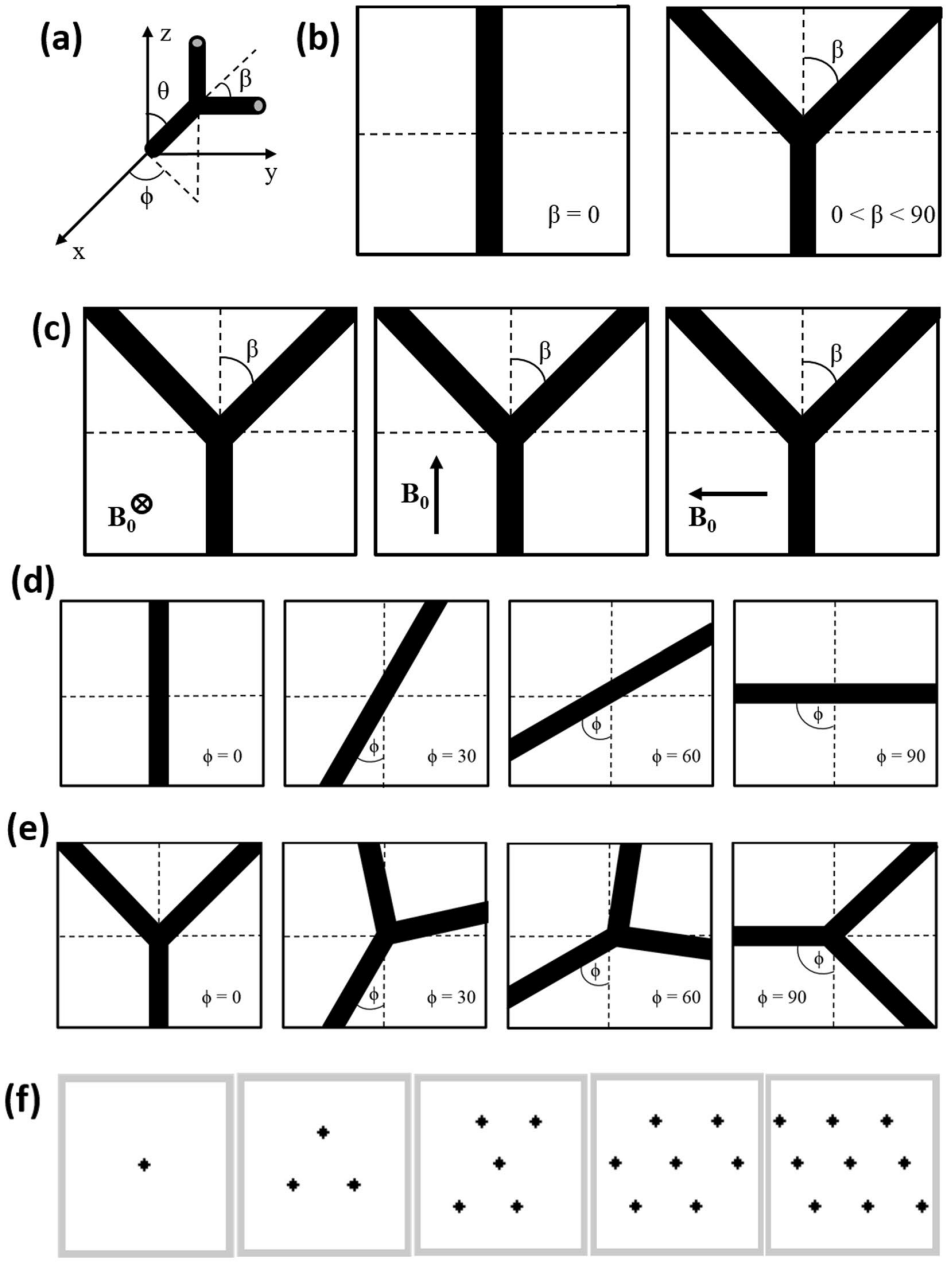
Cylinder fork model design. (**a**) Axis orientation depicting angles θ, β, and ϕ. (**b**) Depiction of fork cylinders with different bifurcation angles β. (**c**) Orientation of the cylinder fork (β = 45°) with respect to the magnetic field **B**_**0**_ shown in three different directions: into the page (Orientation 1), top of the page (Orientation 2), and to the left (Orientation 3). Depiction of fork cylinders at different ϕ angles with (**d**) β = 0°, and (**e**) β = 45°. (**f**) Arrangement of straight cylinders (β = 0°) shown in 128 × 128 cross-sections.

In this study, we present a basic capillary design model that reflects the morphological angular characteristics of cortical vasculature and can potentially be used for comparing vessels in normal and tumorous tissues. Contrary to conventional infinite cylinder design, we propose a cylinder fork design as the basic element that captures the essence of curvature and tortuosity of vessels with the following adjustable parameters (see Fig. 1): (i) bifurcation angle – angle formed after the split of a straight vessel into two branches; (ii) rotation angle – extent of rotational movement of vessels on the plane of the cylinder fork relative to the magnetic field, and; (iii) orientation of vessels with respect to the magnetic field. Here, we attempted to quantitate the cylinder fork design using each of the aforementioned parameters and investigated their effects on R2* and R2. We also investigated the effects of vessel size, diffusion rate, and susceptibility, within a range of physiological values, on R2* and R2 relationship with the bifurcation and rotation angles.

## Methods

Monte Carlo methods were used to quantify R2* and R2 for cylindrical fork perturbers with different bifurcation and rotation angles, vessel sizes, susceptibility values, orientations relative to magnetic field, and diffusion rates of protons. Scripts in MATLAB (The Mathworks, Inc., Natick, MA) were used to generate all aspects of this simulation study.

### Cylinder Fork Model

The vasculature was modeled using a cylinder fork model (CFM) composed of a straight trunk (prior to bifurcation) of at least half cube length with the bifurcating segments emanating in the other half of the cube. The cube was designed with 64 µm sides and incorporated cylinder fork segments (Fig. 1f shows 1, 3, 5, 7 and 9 segments at bifurcation angle β = 0°, i.e. straight cylinder segments) with varying bifurcation (β = 0°, 15°, 30°, 45°, 60°, 75°, 90°) and rotation (ϕ = 0°, 15°, 30°, 45°, 60°, 75°, 90°) angles that were arranged close to symmetry without any overlapping vessels (Supplementary Fig. S1 shows vessel geometry for different combinations of rotation and bifurcation angles). The angle θ (Fig. 1a) between the trunk and the magnetic field was 90° for Orientations 1 and 3 with the vessel lying in the xy-plane (Fig. 1c), while θ = 0° for Orientation 2 (with the vessel lying along the z-axis). For all the orientations and with multiple forks in the same cube, the cylinder trunks were set in a parallel orientation. This model was converted into a cubic 128 × 128 × 128 matrix with each cylinder element assigned a susceptibility value χ, which is equivalent to the difference between the susceptibility values inside the vessel and the surrounding medium.

### Monte Carlo Simulations

Magnetic field perturbations were calculated using a forward 3D Fourier transform of the susceptibility distribution of the CFM^21,26^ which allowed faster computation of the field variations. Signal generation was initiated by placing a proton at a random location outside the vessels to simulate extravascular signal. Each proton took 50 steps between a pair of 90° and 180° pulses distributed using a Gaussian distribution with zero mean and standard deviation $\sigma =\sqrt{2\cdot D\cdot {\rm{\Delta }}t}$, where *D* is the diffusion rate and each time step Δ*t* = 0.1 ms. This one-dimensional step was repeated along the orthogonal directions to generate the displacement vector. The random walk of 40,000 protons was maintained outside the vessels assuming impermeability of the CFM. The phase accumulated by each proton during every step was calculated as γ·Δ*B*·Δ*t* where γ is the gyromagnetic ratio and Δ*B* is the calculated magnetic field from the susceptibility map.

### Simulation Parameters

The simulations were performed with the following parameters based on previously reported studies^2–4,19,27,28^: (i) true vessel diameters of 2.8, 5.6 and 8.6 µm; (ii) diffusion rates (*D*) of 1 × 10^−9^, 1 × 10^−10^ and 1 × 10^−11^ m^2^/s; (iii) susceptibility values (χ) of 3 × 10^−8^, 1 × 10^−7^, and 4 × 10^−7^ in cgs units (or multiplied by 4π for SI units).

### R2* and R2 Calculation

R2* and R2 were calculated by linear least-square fitting of log signal intensity versus volume fraction. The R-squared values of all fittings were very high (~0.9). Relaxation rates per volume fraction unit were used to remove the dependence on vessel lengths and emphasize the role of the bifurcation and rotation angles (β and ϕ) on R2* and R2 while comparing the different simulation parameters.

### Statistics

Two-way analysis of variance (ANOVA) test was performed to check for any significant effect of β and ϕ on the relaxation rates for the various simulation parameters. A value of *p* < 0.05 was considered significant. All *p*-values corresponding to the ANOVA test for each graph are indicated in the respective graph inlets in all figures. Detailed statistical information are presented in the Supplemental Information for each figure.

### Data availability

The datasets generated and analyzed during this study are available from the corresponding author on reasonable request.

## Results

### Effect of vessel size on R2* and R2 relationship with the bifurcation angle at different orientations and rotation angles

**Figure 2:**
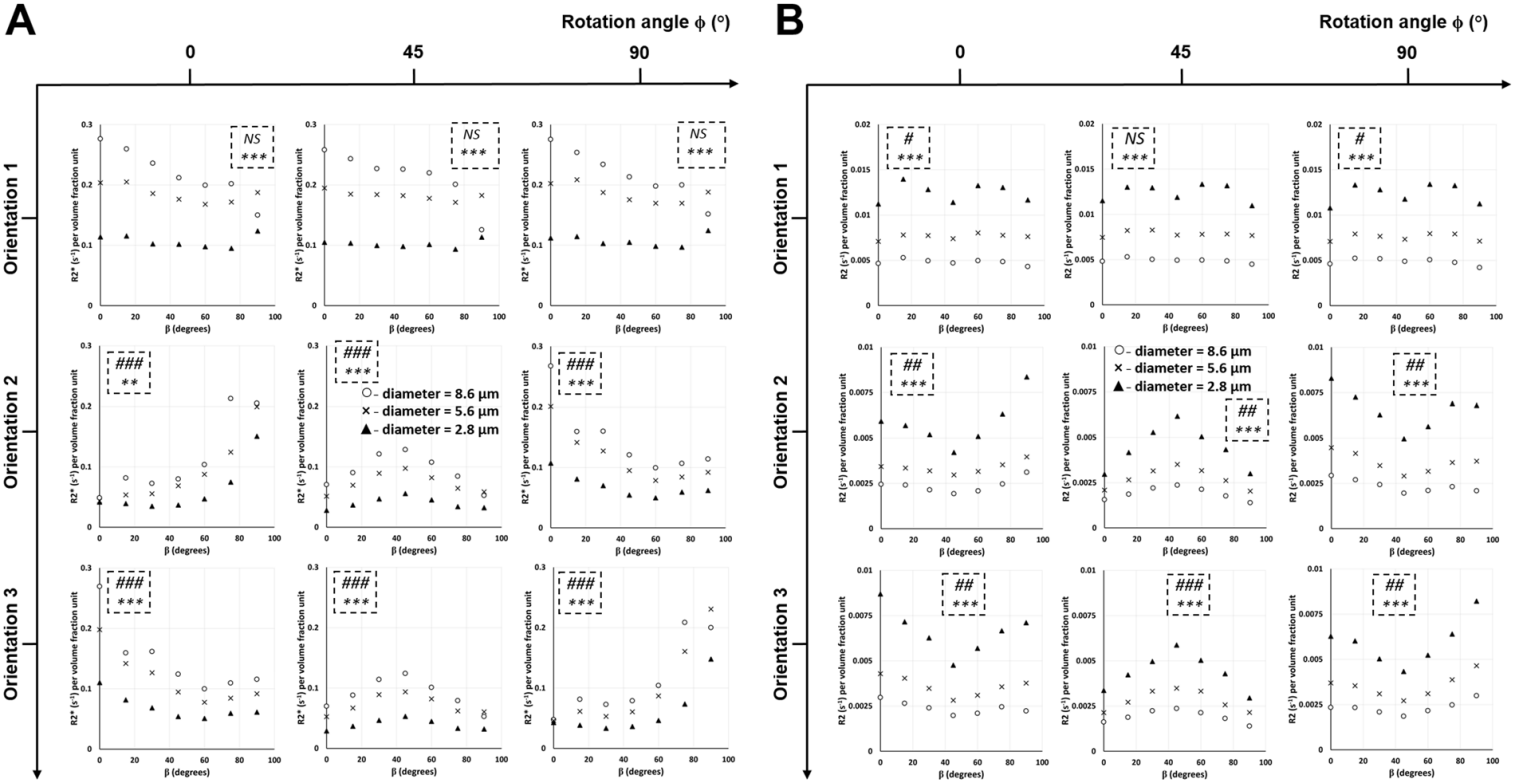
Vessel size effect on R2* and R2 relationship with the bifurcation angle. Plots of R2* (**A**) and R2 (**B**) per volume fraction unit as a function of the bifurcation angle β are shown with three different vessel diameters at three different orientations and rotation angles. The diffusion rate and susceptibility value are 1 × 10^−10^ m^2^/s and 3 × 10^−8^ in cgs units, respectively. The statistical significances from a two-way ANOVA are shown for each graph in the dotted inlet, where the top (#) and bottom (*) symbols show the significant effects of bifurcation angle and vessel diameter, respectively, on R2* and R2. # −0.01 < *p* < 0.05; ##, ** −0.001 < *p* < 0.01; ###, *** −*p* < 0.001; NS – not significant. Detailed statistical values are shown in Supplementary Table S1.

Figure 2A (extended version in Supplementary Fig. S2) shows the effect of vessel size on R2* relationship with the bifurcation angle. An increase in vessel size caused an increase in R2* for all orientations and rotation angles. In Orientation 1 (i.e. θ = 90°), R2* showed a decreasing trend as the bifurcation angle (β) was increased for all rotation angles (ϕ). Increasing the rotation angles between 0° and 90° slightly oscillated R2* values in Orientation 1. Orientations 2 and 3 showed a mirror effect of R2* vs. β with respect to rotation angles: in Orientation 2, R2* showed (i) an increasing trend with an increase in bifurcation angle between rotation angles of 0° and 45°, and (ii) a decreasing trend with an increase in bifurcation angle between rotation angles of 45° and 90°. The exact opposite of Orientation 2 was observed in Orientation 3. At the rotation angle of 45°, Orientations 2 and 3 looked very similar, where R2* showed a symmetry with an inverted V-profile around the bifurcation angle of 45° for all vessel sizes. ANOVA results showed: (i) significant effect of vessel size on R2* for all orientations and rotation angles, and; (ii) significant effect of bifurcation angle on R2* in all rotation angles for Orientations 2 and 3, but not in Orientation 1.

Figure 2B (extended version in Supplementary Fig. S3) shows the effect of vessel size on R2 relationship with the bifurcation angle. Unlike R2*, an increase in vessel size caused a decrease in R2 for all orientations and rotation angles. In Orientation 1, R2 showed symmetry with an M-profile around the bifurcation angle of 45° which became less pronounced with larger vessel sizes. Increasing the rotation angles between 0° and 90° slightly oscillated the R2 values in Orientation 1. In Orientation 2, R2 vs. β showed a change in concavity from concave upward (V-profile) to concave downward (inverted V-profile) to concave upward (V-profile) between rotation angles of 0°, 45°, and 90°, respectively. Orientation 3 showed a similar trend of concavity for the R2 vs. β plots with respect to the rotation plots of Orientation 2. However, similar to the pattern for R2*, Orientations 2 and 3 showed a mirror effect of R2 vs. β with respect to the rotation angles: the V-profile pattern in Orientation 2 from 0° to 90° resembled that of Orientation 3 from 90° to 0°. At the rotation angle of 45°, Orientations 2 and 3 looked very similar, where R2 showed a symmetry with an inverted V-profile around the bifurcation angle of 45° for all vessel sizes, similar to the pattern seen with R2*. ANOVA results showed: (i) significant effect of vessel size on R2 for all orientations and rotation angles, and; (ii) significant effect of bifurcation angle on R2 in all rotation angles for Orientations 2 and 3; in Orientation 1, all rotation angles except 45° showed a significant effect.

### Effect of vessel size on R2* and R2 relationship with the rotation angle at different orientations and bifurcation angles

Data in Fig. 2 can be re-drawn to show the effect of vessel size on R2* and R2 relationship with the rotation angle at different orientations and bifurcation angles (extended version in Supplementary Fig. S4). ANOVA results showed: (i) significant effect of vessel size on R2* for all orientations and bifurcation angles, and; (ii) significant effect of rotation angle on R2* in most bifurcation angles for all orientations.

Similar ANOVA analysis was performed with regard to the effect of vessel size on R2 relationship with the rotation angle, which reveals that: (i) a significant effect of vessel size on R2 was observed for all orientations and bifurcation angles, and; (ii) a significant effect of rotation angle on R2 was observed in most bifurcation angles for Orientations 2 and 3; orientation 1 did not show significant effects for most bifurcation angles.

### Effect of diffusion rate on R2* relationship with the rotation angle at different bifurcation angles, vessel sizes, and susceptibility values

**Figure 3:**
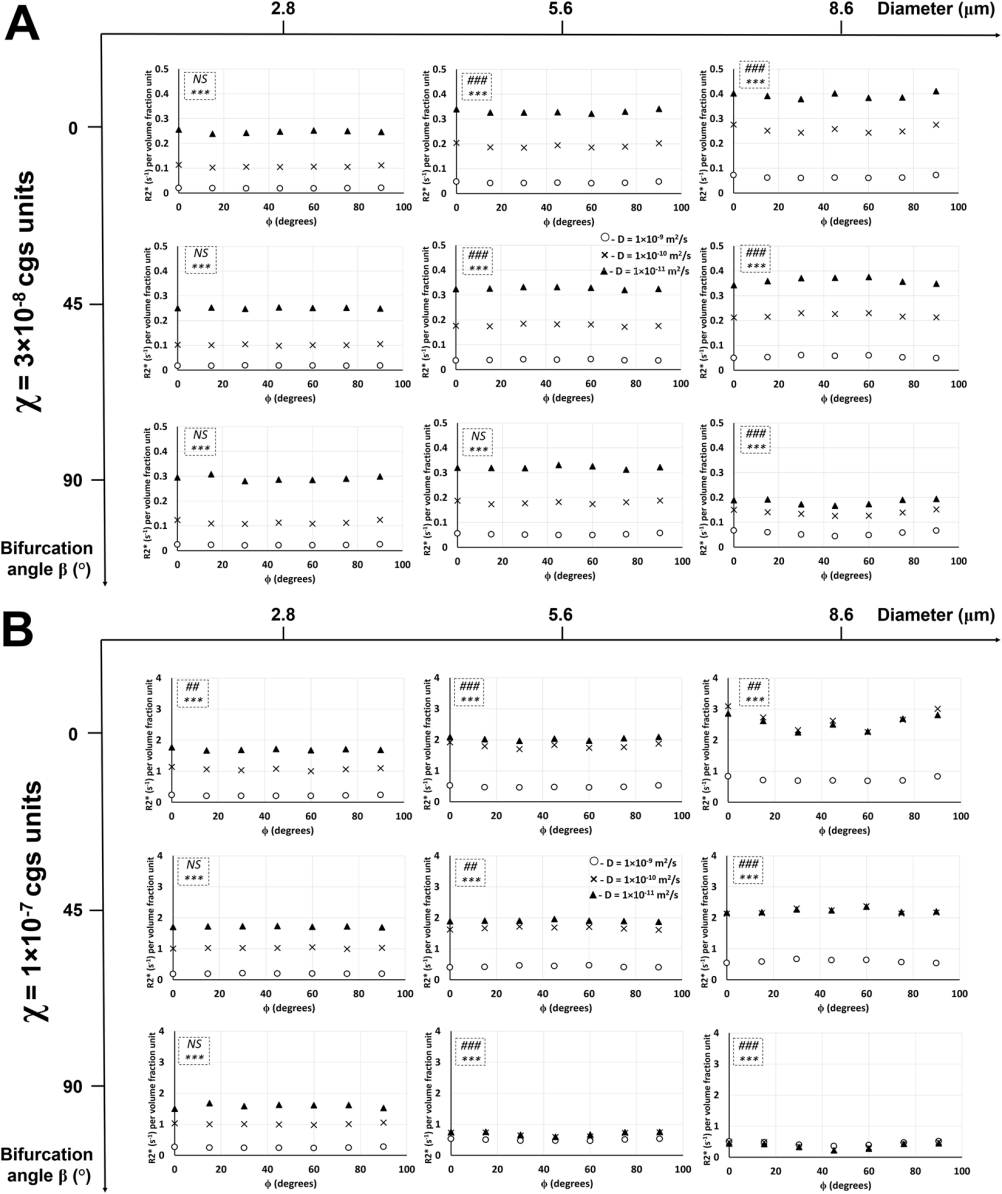
Diffusion rate effect on R2* relationship with the rotation angle in Orientation 1. Plots of R2* per volume fraction unit as a function of the rotation angle ϕ in Orientation 1 and susceptibility values χ = 3 × 10^−8^ cgs units (**A**) and χ = 1 × 10^−7^ cgs units (**B**) are shown with three different diffusion rates at three different bifurcation angles and vessel diameters. The statistical significances from a two-way ANOVA are shown for each graph in the dotted inlet, where the top (#) and bottom (*) symbols show the significant effects of rotation angle and diffusion rate on R2*, respectively. ## −0.001 < *p* < 0.01; ###, ***p < 0.001; NS – not significant. Detailed statistical values are shown in Supplementary Table S2.

**Figure 4:**
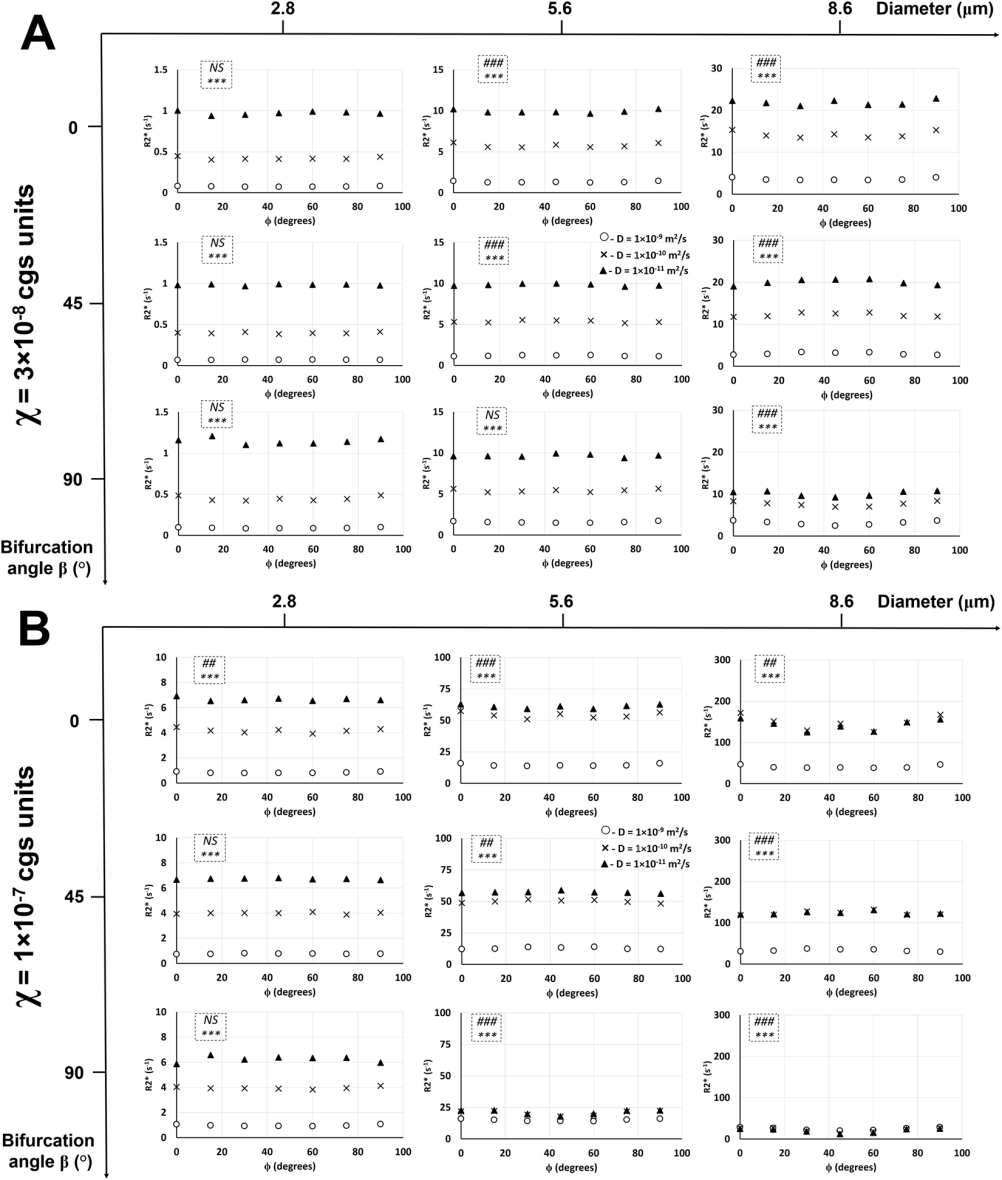
Diffusion rate effect on R2* relationship with the rotation angle in Orientation 1 without normalization. Same plots from Fig. 3 are shown here with all normalizations removed. Plots of R2* as a function of the rotation angle ϕ in Orientation 1 and susceptibility values χ = 3 × 10^−8^ cgs units (**A**) and χ = 1 × 10^−7^ cgs units (**B**) are shown with three different diffusion rates at three different bifurcation angles and vessel diameters. The statistical significances from a two-way ANOVA are shown for each graph in the dotted inlet, where the top (#) and bottom (*) symbols show the significant effects of rotation angle and diffusion rate on R2*, respectively. ## −0.001 < *p* < 0.01; ###, ***p < 0.001; NS – not significant. Detailed statistical values are shown in Supplementary Table S3.

Figure 3 shows the effect of diffusion rate on R2* relationship with the rotation angle in Orientation 1 (same effect for Orientation 3 in Supplementary Fig. S6). Figure 4 shows the same plots in Fig. 3, but without the normalization by vessel length and area. An increase in diffusion rate caused a decrease in R2* for all vessel sizes and bifurcation angles in both susceptibility value cases. An increase in vessel size caused a slight increase in R2* vs. ϕ plots for all diffusion rates and all bifurcation angles except in the case of the largest vessel size (Fig. 3A) and two larger vessel sizes (Fig. 3B) at the bifurcation angle of 90°, where the R2* values decreased for the two lower diffusion rates. Increasing the vessel size also reduced the R2* gap between the two lower diffusion rates and increased the R2* gap between the lower two diffusion rates and the highest one, which is more vivid at the higher susceptibility value (χ = 1 × 10^−7^). At this higher susceptibility value, in addition to increased R2* values for all cases, R2* vs. ϕ plots overlapped very closely for the two lower diffusion rates at the two larger vessel sizes at bifurcation angles of 0° and 45°. At a bifurcation angle of 90°, R2* vs. ϕ plots for all three diffusion rates overlapped each other for the two larger vessel sizes. ANOVA results showed: (i) significant effect of diffusion rate on R2* for all vessel sizes and bifurcation angles at both susceptibility values, and; (ii) significant effect of rotation angle on R2* at both susceptibility values for most of the two larger vessel sizes; the smallest vessel size did not show significance for most of the cases.

### Effect of diffusion rate on R2* relationship with the bifurcation angle at different rotation angles, vessel sizes, and susceptibility values

**Figure 5:**
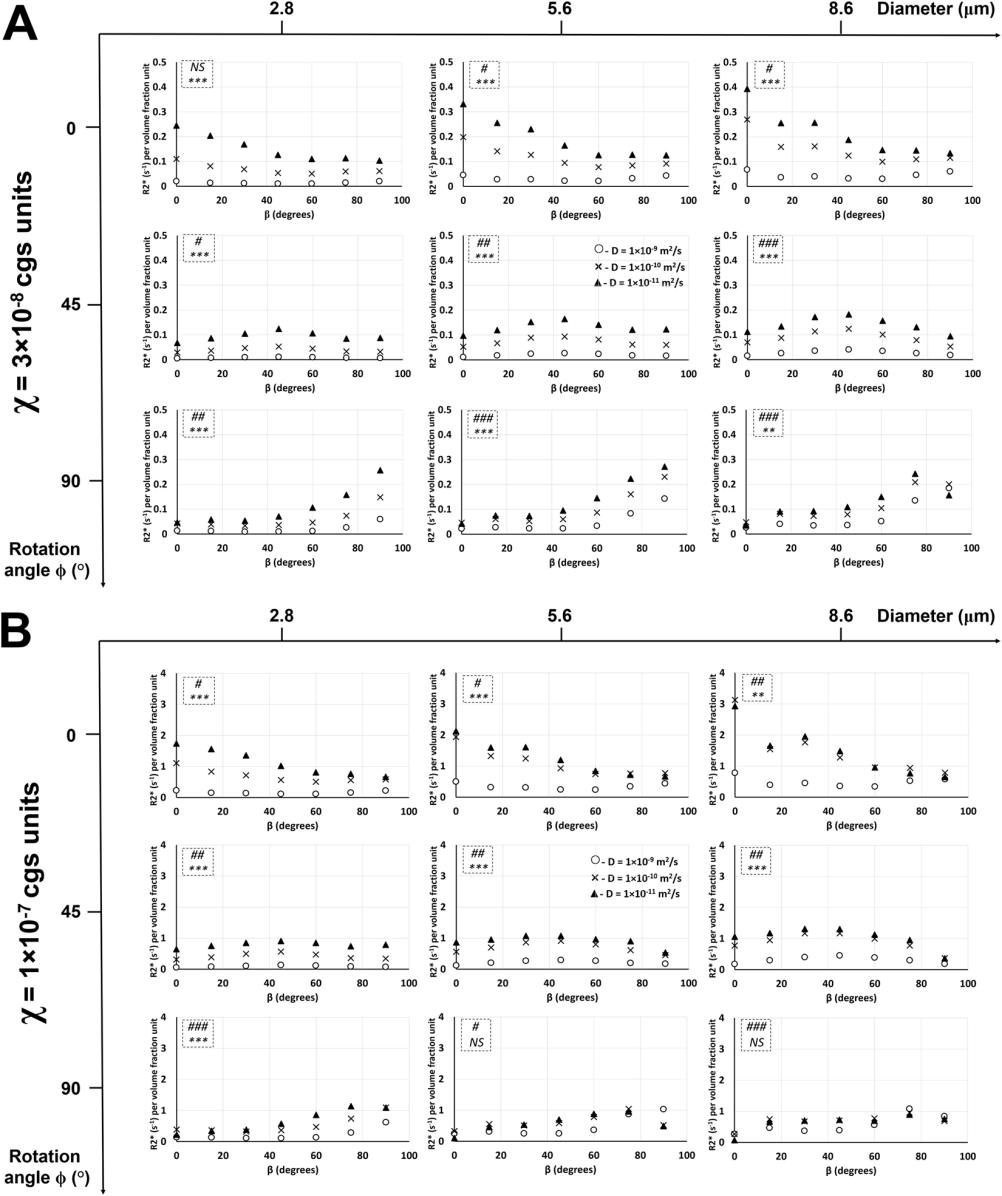
Diffusion rate effect on R2* relationship with the bifurcation angle in Orientation 3. Plots of R2* per volume fraction unit as a function of the bifurcation angle β in Orientation 3 and susceptibility values χ = 3 × 10^−8^ cgs units (**A**) and χ = 1 × 10^−7^ cgs units (**B**) are shown with three different diffusion rates at three different rotation angles and vessel diameters. The statistical significances from a two-way ANOVA are shown for each graph in the dotted inlet, where the top (#) and bottom (*) symbols show the significant effects of bifurcation angle and diffusion rate on R2*, respectively. # −0.01 < *p* < 0.05; ##, ** −0.001 < *p* < 0.01; ###, ***p < 0.001; NS – not significant. Detailed statistical values are shown in Supplementary Table S4.

Figure 5 shows the effect of diffusion rate on R2* relationship with the bifurcation angle in Orientation 3 (same effect for Orientation 1 in Supplementary Fig. S7). An increase in diffusion rate caused a decrease in R2* for all vessel sizes and rotation angles in both orientations and both susceptibility value cases. For both Orientations 1 and 3 and in both susceptibility value cases, an increase in vessel size caused a slight increase in R2* vs. β plots for all diffusion rates and all rotation angles. Increasing the vessel size also reduced the R2* gap between the lower two diffusion rates and increased the R2* gap between the lower two diffusion rates and the highest one, which is more vivid at the higher susceptibility value (χ = 1 × 10^−7^). In both Orientations 1 and 3, increasing the susceptibility value caused an increase in R2* values for all cases. At the higher susceptibility value, R2* vs. β plots overlapped very closely for the two lower diffusion rates and two larger vessel sizes at all rotation angles in both orientations. In Orientation 3 at the rotation angle of 90°, R2* vs. β plots for all three diffusion rates overlapped each other for the two larger vessel sizes. ANOVA results showed: (i) significant effect of diffusion rate on R2* for all vessel sizes and bifurcation angles at both susceptibility values in both orientations except for the higher susceptibility value in Orientation 3 with the two larger vessel sizes at the rotation angle of 90°, and; (ii) significant effect of bifurcation angle on R2* at both susceptibility values for most of the two larger vessel sizes in both orientations; for the smallest vessel size, however, more significances were observed in Orientation 3 compared to Orientation 1 for most rotation angles at both susceptibility values.

### Effect of diffusion rate on R2 relationship with the rotation and bifurcation angles at different susceptibility values and vessel sizes

**Figure 6:**
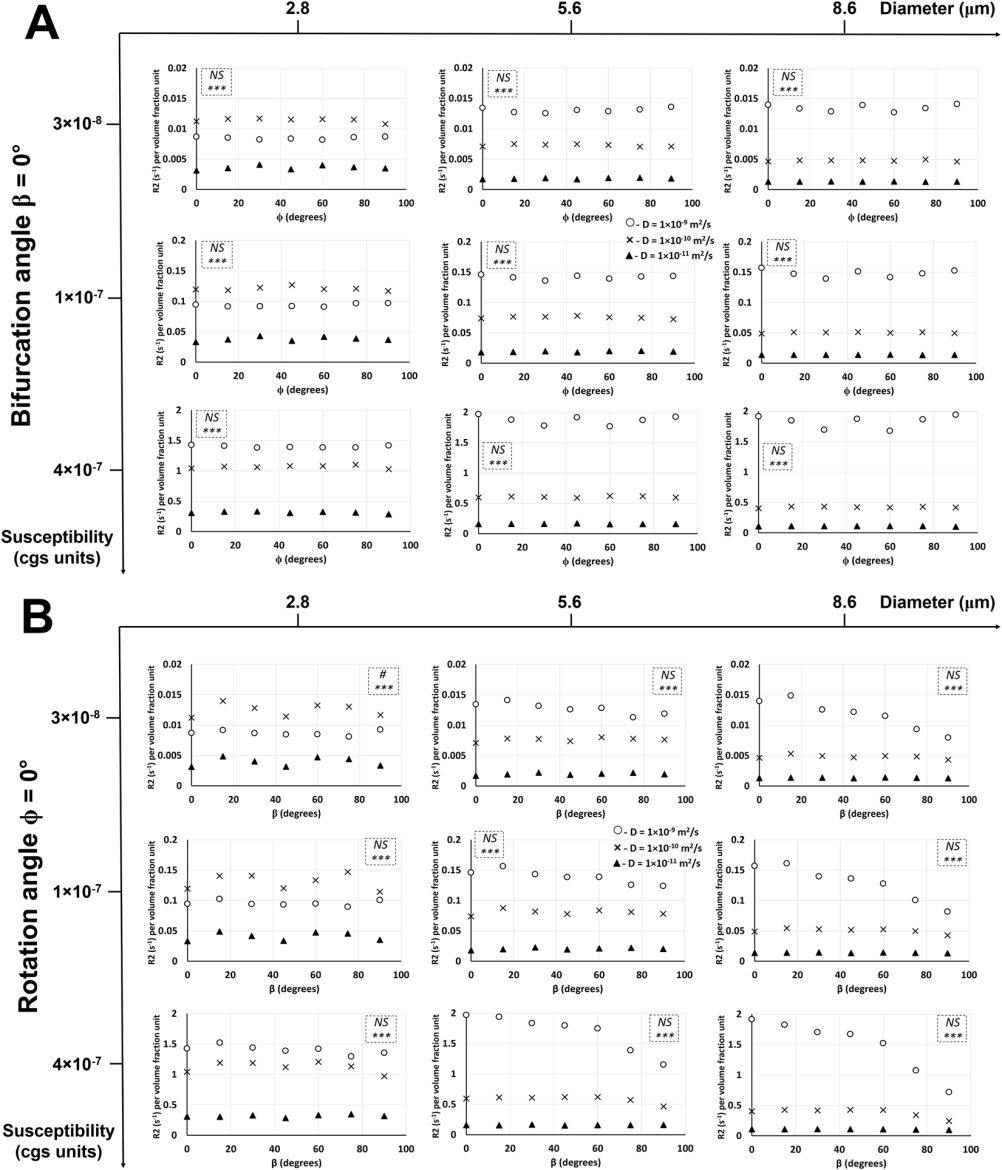
Diffusion rate effect on R2 relationship with the rotation and bifurcation angles in Orientation 1. (**A**) Plots of R2 per volume fraction unit as a function of the rotation angle ϕ in Orientation 1 and bifurcation angle β = 0° are shown with three different diffusion rates at three different susceptibility values and vessel diameters. The statistical significances from a two-way ANOVA are shown for each graph in the dotted inlet, where the top and bottom (*) symbols show the significant effects of rotation angle and diffusion rate on R2, respectively. *** −*p* < 0.001; NS− not significant. (**B**) Plots of R2 per volume fraction unit as a function of the bifurcation angle β in Orientation 1 and rotation angle ϕ = 0° are shown with three different diffusion rates at three different susceptibility values and vessel diameters. The statistical significances from a two-way ANOVA are shown for each graph in the dotted inlet, where the top (#) and bottom (*) symbols show the significant effects of bifurcation angle and diffusion rate on R2, respectively. # −0.01 < *p* < 0.05; *** −*p* < 0.001; NS – not significant. Detailed statistical values are shown in Supplementary Table S5.

**Figure 7:**
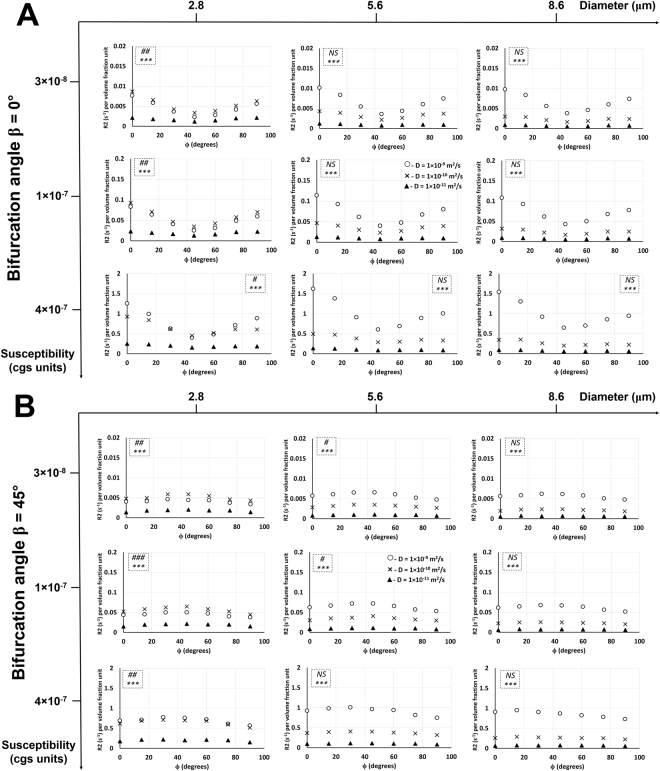
Diffusion rate effect on R2 relationship with the rotation angle in Orientation 3. Plots of R2 per volume fraction unit as a function of the rotation angle ϕ in Orientation 3 and bifurcation angle β = 0° (**A**) and β = 45° (**B**) are shown with three different diffusion rates at three different susceptibility values and vessel diameters. The statistical significances from a two-way ANOVA are shown for each graph in the dotted inlet, where the top (#) and bottom (*) symbols show the significant effects of rotation angle and diffusion rate on R2, respectively. # −0.01 < *p* < 0.05; ## −0.001 < *p* < 0.01; ###, *** −*p* < 0.001; NS – not significant. Detailed statistical values are shown in Supplementary Table S6.

**Figure 8:**
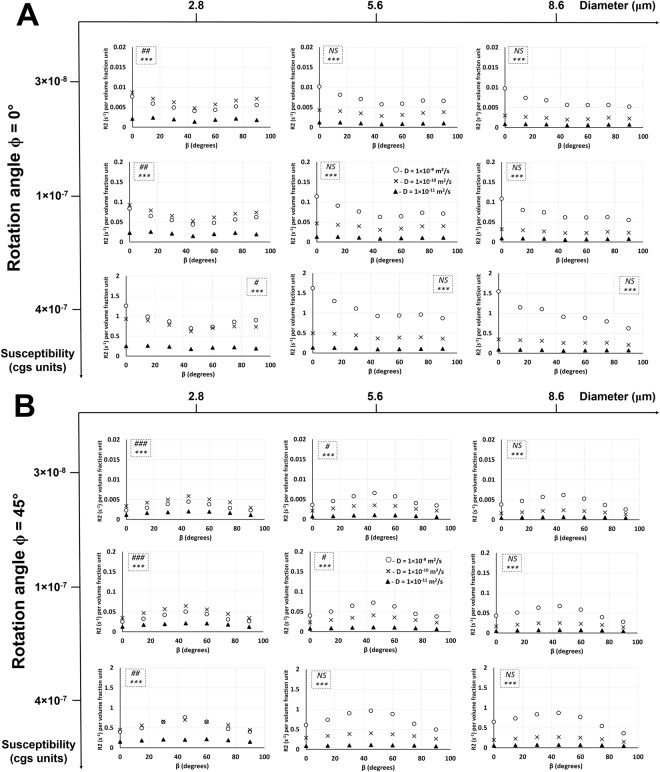
Diffusion rate effect on R2 relationship with the bifurcation angle in Orientation 3. Plots of R2 per volume fraction unit as a function of the bifurcation angle β in Orientation 3 and rotation angle ϕ = 0° (**A**) and ϕ = 45° (**B**) are shown with three different diffusion rates at three different susceptibility values and vessel diameters. The statistical significances from a two-way ANOVA are shown for each graph in the dotted inlet, where the top (#) and bottom (*) symbols show the significant effects of bifurcation angle and diffusion rate on R2, respectively. # −0.01 < *p* < 0.05; ## −0.001 < *p* < 0.01; ###, *** −*p* < 0.001; NS− not significant. Detailed statistical values are shown in Supplementary Table S7.

Figure 6 shows, in Orientation 1, the effect of diffusion rate on R2 relationship with the rotation angle at a bifurcation angle of 0° and R2 relationship with the bifurcation angle at a rotation angle of 0°. Figures 7 and 8 show, in Orientation 3, the effect of diffusion rate on R2 relationship with the rotation angle at bifurcation angles of 0° and 45° and R2 relationship with the bifurcation angle at rotation angles of 0° and 45°, respectively. An increase in diffusion rate caused an increase in R2 with respect to the rotation and bifurcation angles for all vessel sizes and susceptibility values except for the lowest vessel size at the two lower susceptibility values: in these cases, increasing the diffusion rate from 1 × 10^−11^ m^2^/s to 1 × 10^−10^ m^2^/s caused an increase in R2 values, but then an increase from 1 × 10^−10^ m^2^/s to 1 × 10^−9^ m^2^/s caused a decrease in R2 values. An increase in susceptibility values caused an increase in R2 values for all cases. For both R2 vs. ϕ and R2 vs. β plots and for all susceptibility values in both Orientations 1 and 3, an increase in vessel size caused a general increase in R2 values for the highest diffusion rate. The two lower diffusion rates showed a decrease in R2 values with increasing vessel size. Increasing the vessel size also increased the R2 gap between the highest diffusion rate and the lower two diffusion rates. R2 gap between the two lower diffusion rates decreased with increasing vessel size. ANOVA results showed: (i) significant effect of diffusion rate on R2 for all vessel sizes and susceptibility values for both bifurcation and rotation angles in both orientations; (ii) no significant effect of the rotation angle on R2 in Orientation 1; however, in Orientation 3, significance was mainly observed at the smallest vessel size, and; (iii) no significant effect of the bifurcation angle on R2 in Orientation 1 except at the smallest vessel size and lowest susceptibility value; however, in Orientation 3, significance was mainly observed at the smallest vessel size.

### Summary of R2* and R2 relationship with the correlation time at different orientations and susceptibility values

**Figure 9:**
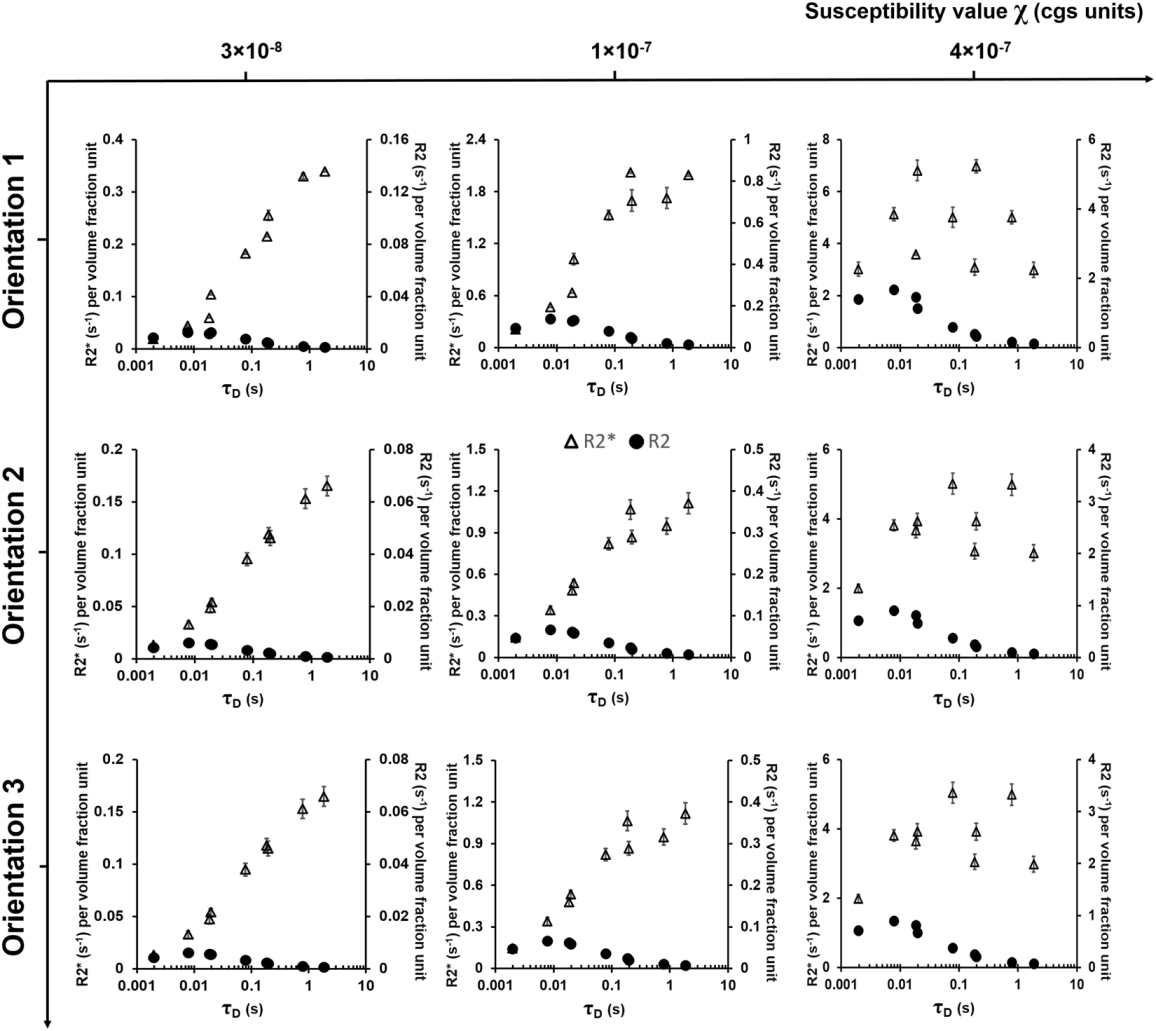
R2* and R2 relationship with the correlation time using the CFM. Plots of R2* (left axis) and R2 (right axis) per volume fraction unit as a function of correlation time (*τ*_*D*_) are shown in three different orientations and at three different susceptibility values. The R2* and R2 values shown are the mean averages taken across all the bifurcation (β = 0°, 15°, 30°, 45°, 60°, 75°, 90°) and rotation (ϕ = 0°, 15°, 30°, 45°, 60°, 75°, 90°) angles. The error bars on each data point indicate the standard error mean (SEM).

Figure 9 shows a summary of R2* and R2 relationship with the correlation time (*τ*_*D*_) in Orientations 1, 2, and 3 and at susceptibility values of 3 × 10^−8^, 1 × 10^−7^, and 4 × 10^−7^ cgs units. The plotted R2* and R2 values are the mean values taken across all the bifurcation and rotation angles (β, ϕ = 0°, 15°, 30°, 45°, 60°, 75°, 90°) to show the overall effect of *τ*_*D*_ on R2* and R2. Orientations 2 and 3 show nearly identical profiles. When *τ*_*D*_ is short (<0.01 s), little difference is seen between R2* and R2. As *τ*_*D*_ is increased, R2 reaches a peak and then begins to decrease, while R2* keeps increasing and approaches a static limit. With increasing susceptibility: (i) R2 peak value increases and shifts slightly to the left in all three orientations, and; (ii) R2* values increase with more fluctuation at higher *τ*_*D*_ values near the static limit, more so in Orientation 1 compared to Orientations 2 and 3. These fluctuations are due to the averaging of R2* over different bifurcation and rotation angles.

## Discussion

When simulating BOLD contrast effects in fMRI, most researchers make the general assumption that the brain vasculature is represented as 2D or 3D infinite long cylinders to simulate BOLD signal changes^29^. The CFM structure presented in this study captures the basic angular characteristics of vascular architecture using the vessel orientation relative to the magnetic field, and the vessel bifurcation and rotation angles. To understand the effect of each of these angular variations on R2* and R2, vessel forks were simulated in different orientations and modelled with respect to realistic cortical vasculature measurements.

Vessel diameters were chosen in the capillary range from ~2‒10 µm based on morphometric data from normal tissue and C_6_ astrocytoma cells that were implanted in rats^19^. This diameter range covered the lumen diameters that were observed in normal, peritumoral, and tumor microvessels both in the brain and muscle tissue^19^. These values also agree with the lower-end vessel diameters observed in different brain tumor grades in patients^30^. For computation efficiency and fitting the vessel size with the appropriate volume fraction in the voxel space, 128 × 128 × 128 matrix was used. The susceptibility values were chosen between 0 and 0.6 × 10^−6^ cgs units to cover a wide range of biologically relevant contrast agent concentrations^2,4^. The largest diffusion rate of 1 × 10^−9^ m^2^/s was chosen based on the typical value reported for the cerebral cortex^27,28^. The other two lower diffusion rates span the range of expected diffusion rates in tissue^3^.

Bifurcation and rotation angles were chosen between 0° and 90° to simplify the model, since larger angles would yield predictable results based on the 0°‒90° range. The three orientations studied in this model covered all three dimensions of the vessel topography. Depending on the chosen geometry, Orientations 2 and 3 showed almost a mirror effect for R2* and R2 with the bifurcation and rotation angles (Figs 2 and S2‒S5); hence Orientation 3, in addition to Orientation 1, was used in most of the illustrations in this simulation study. Also, the variation of R2* and R2 with the vessel diameter was opposite to each other in all orientations (Figs 2 and S2‒S5) because R2* lies within the MAR for the two smaller diameters and just shifts to the SDR limit for the largest diameter. However, R2 decreased with increasing diameter since that range falls within the ELR. Both patterns depend on the values used for the diffusion rates and susceptibility.

R2* and R2 showed an increase with the vessel size (or *τ*_*D*_) within the MAR up to *τ*_*D*_ = 0.01 s (Fig. 9). R2* reached a plateau only for the two higher susceptibility values while R2 showed a decrease with vessel size after *τ*_*D*_ = 0.01 s: this is indicative of the fact that R2 lies within the ELR. These R2* and R2 curves are very similar to Fig. 2 from Boxerman *et al*.^2^ and Fig. 3 from Weisskoff *et al*.^4^ which validates the simulations conducted with the CFM. For a small vessel radius or *τ*_*D*_, both R2* and R2 are equal because of the efficiency of fast diffusion to average field inhomogeneities^21,23^. Thereafter, R2* reaches a plateau while R2 decreases if the echo-time is small compared to *τ*_*D*_. This is seen to occur at *τ*_*D*_ ≈ 10 ms, which agrees with the condition for ELR using our value for *τ*_*CP*_ = 5 ms.

The R2* and R2 patterns observed in all the scenarios presented in this study are an outcome of the resultant field inhomogeneities caused due to the interaction of the respective vessel topology with the B_0_ magnetic field. For example, in Orientation 2 (where θ = 0°), at a rotation angle ϕ = 0°, the vessel exhibits a T-shape when the bifurcation angle β = 90°. This scenario presents the maximum disturbance in the local field caused by the interaction of B_0_ and vessel geometry, hence yielding larger R2* and R2 values (Figs 2 and S2‒S5). We can consider the form of the magnetic field outside the vessel (e.g. Eq. 1 from Boxerman *et al*.^2^):

6 $${\rm{\Delta }}B={B}_{0}2\pi {\rm{\Delta }}\chi {(\frac{R}{r})}^{2}\,\cos (2\phi )\,si{n}^{2}\theta $$

where Δχ is the susceptibility difference between the intra- and extra-cylindrical space, *R* is the vessel radius, *r* is the distance of proton from the cylinder axis, and φ is the rotation angle ϕ from our nomenclature. For a bifurcation angle β = 90° (at θ = φ = 0°), a contribution from the fork segments to the orientation angle θ will be made through the *sin* ^2^ *θ* term while the trunk segment will be oriented at θ = 0°, and hence making no contribution. The total contribution will, therefore, be non-zero unlike the case for a straight segment (i.e., β = 0°).

Care must be exercised when interpreting Fig. 3 since the bifurcation angle has an effect on the volume fraction due to the normalization performed in this study. The length of the vessel (e.g. at θ = 0°) for the angles β = 0°, 45°, and 90° is *2a*, *a*∙(1 + 2√2), and *3a*, respectively, where *2a* is the voxel size. Another normalization process takes place with respect to the diameter of the vessel which must also be taken into account for our results. When normalization is removed (as is routinely presented) from the data of Fig. 3, then Fig. 4 shows that R2* is consistent with the MAR (Eq. 3) for all diameter, diffusion rate, and susceptibility values.

The larger two vessel sizes showed more significant effects of bifurcation and rotation angles on R2* compared to the smallest vessel size in both the susceptibility values in Orientation 1 (Figs 3 and S7). Since the peritumoral and tumor microvessels fall in this range of vessel size, the bifurcation and rotation angles can potentially be used as a distinguishing factor in delineating these vessels from normal vasculature when the vessels are oriented in this particular alignment. In Orientation 3, nearly all the vessel sizes showed a significant effect of bifurcation and rotation angles on R2* for both susceptibility values (Figs 5 and S6). However, for the larger two vessel diameters at the higher susceptibility value, the diffusion rates did not show a significant effect on R2* (Figs 5B and S6B) which is contrary to Orientation 1, where the diffusion rates showed a significant effect on R2* for all cases. The application of contrast agent for vessels oriented in the Orientation 3 configuration might be useful in distinguishing vessels that have T-shape bifurcations which is more common in cases of tumor vasculature.

Unlike R2*, in Orientation 1, nearly all the vessel sizes did not show a significant effect of the bifurcation and rotation angles on R2 for all susceptibility cases (Fig. 6). In Orientation 3, only the lower vessel sizes showed a significant effect of bifurcation and rotation angles on R2 (Figs 7 and 8). Thus, when combined with the information from the R2* experiments, the larger vessels can be distinguished from the lower ones based on the bifurcation and rotation angle signature given by the R2* and R2 values. Another interesting point to note is that at the smallest vessel size and the two lower susceptibility values, the R2 values increased with the diffusion rate and then decreased again (Figs 6, 7 and 8), while the R2* values for the respective scenario just decreased with increasing diffusion rates (Figs 3, 5, S6 and S7). This behavior is indicative of a shift of R2 from the MAR towards the ELR^2^. This indicates that the transition from the MAR into the ELR (for the smallest vessel diameter and intermediate *D* values), which occurs at *∆ω*∙*τ*_*D*_ ≈ 1, is satisfied for *∆ω* = 51 rad/s, a value consistent with susceptibility values. The agreement of the transverse relaxation rates behavior with the correlation time using the CFM compared to previous studies^2,4^, further validates the use of this model to develop realistic cortical vasculature patterns.

In summary, R2* and R2 measurements indicated a clear dependence on the bifurcation and rotation angles in scenarios using different orientations, diffusion rates, susceptibility values and vessel diameters in the capillary range. Presumably, at higher vessel diameters (reflecting higher grade tumors^30^), the differences would be more vivid given that we were able to see differences just within the capillary range. The simulations also demonstrated that at certain bifurcation and rotation angles, the larger vessel sizes showed clear separation between the higher and lower diffusion rates at high vessel susceptibilities. This can potentially reflect a physiological scenario where with larger diameters (tumorous tissue) and increased susceptibility (presence of contrast agent) can delineate the relaxation rates in relation to the bifurcation and rotation angles as being either in the MAR (high *D* value) or in the SDR/ELR (lower *D* values). Since the apparent diffusion coefficient (ADC) is known to be lower, which corresponds to lower diffusion rates, in low-grade tumors^25^, the quantifications presented herein using the CFM could be further explored and exploited to potentially differentiate normal vessels from tumor vessels based on a combination of diffusion rates and bifurcation and rotation angles. Some discussion in regards to model limitations and improvements have been included in the Supplementary Information.

In order to evaluate tumor angiogenesis using MRI techniques, it is absolutely necessary to better understand the role of many morphological, biophysical, and experimental factors. To achieve this goal, computational modeling of susceptibility-induced MR contrast has been employed for a long time^1–8^ relying mainly on using cylindrical or spherical perturbers. However, these simple models may not be adequate to study pathology such as anomalous vascular morphology found in tumors^10,31^. The finite perturber method^10^ was developed towards modeling susceptibility-induced field perturbations emanating from the microvascular (i.e. sub-voxel) level arising from arbitrary microvascular morphologies such as those typically exhibited in angiogenesis. The motivation for such approach was that differences in ∆R2* contrast observed in brain tumor angiogenesis (e.g. the calibration factor between contrast material dose and measured ∆R2* values)^32,33^ can be due to different vascular morphology of brain tumors compared to healthy brain tissue (i.e. not necessarily due to differences in fractional vascular volumes)^34^. Abnormal vascular phenotypes have been implicated in neuropathologies ranging from Alzheimer’s^35^ disease to brain tumors^36,37^ while cerebral vasculature is central to understanding the hemodynamics of novel therapies in the brain^38^.

Our work presents a novel vascular model that attempts to more accurately explain and predict MR signal changes and its critical dependence on 3D vessel architecture. The CFM model uniquely: 1) separates the effects of various vessel-specific morphological parameters (such as length, radius, various vessel-related angles, etc.) through the introduction of the fork concept, and; 2) offers a building block for MR signal source that can be combined with other similar or different blocks to take into account the complex morphology exhibited by a brain tumor. The design of the model uniquely offers a number of adjustable parameters that can be independently optimized to simulate the susceptibility-induced MR contrast. This feature makes the CFM uniquely suited to model complex vessel architecture such as exhibited by tumors. Furthermore, this approach will allow the investigation of an arbitrary and often complex vascular architecture and not be restricted by limitations seen in traditional models such as parallel, infinitely long cylindrical vessels, or non-overlapping magnetic fields.

## Electronic supplementary material

Supplementary Information
