# Supplementary material for: MRI Simulation Study Investigating Effects of Vessel Topology, Diffusion, and Susceptibility on Transverse Relaxation Rates Using a Cylinder Fork Model
Source: Sci Rep. 2017 Nov 24;7:16223. doi: 10.1038/s41598-017-15968-4 (PMC5701222; doi:10.1038/s41598-017-15968-4)
Supplement: Supplementary file 1 — Supplementary Information [file 41598_2017_15968_MOESM1_ESM.pdf]

Supplementary Information:

MRI Simulation Study Investigating Effects of Vessel Topology, Diffusion, and Susceptibility on Transverse Relaxation Rates Using a Cylinder Fork Model

Mohammed Salman Shazeeb[1,2], Jayashree Kalpathy-Cramer[2], Bashar Issa[1]

[1]Department of Physics
College of Science
UAE University
Al-Ain, UAE

[2]Department of Radiology
Athinoula A. Martinos Center for Biomedical Imaging
Massachusetts General Hospital and Harvard Medical School
Boston, MA, USA

Correspondence:
Bashar A. Issa, Ph.D.
Department of Physics
UAE University, P.O.Box 15551
Al-Ain, Abu Dhabi, UAE
Tel : +971 3 713 6316
FAX : +971 3 713 6944
E-mail: b.issa@uaeu.ac.ae

Supplementary Discussion

Model limitations and future work

The CFM models the capillaries as parallel cylinders with bifurcation forks. This model can be further developed to incorporate more realistic vascular networks with larger diameters that would include other shapes and loops in different random orientations to reflect a more realistic vasculature representation. Another feature that can be added to the CFM would be to rotate the fork around the trunk axis. Further improvements can also include intravascular signal in the cylinders to reflect flow within the vessels and their contribution to the relaxation rates in the context of vessel morphology. Another point to note here is that the bifurcation model used in this study assumes vessel branches to maintain the same diameter as the trunk; that is clearly an unrealistic assumption since in reality, the branching of vessels is almost always accompanied by a reduction in vessel size [1,2,3]. However, the emphasis of this study is on morphology in order to develop the basic features that would allow linear combinations of many elements to simulate a realistic capillary pattern.

Supplementary Figures and Tables

Supplementary Figure S1

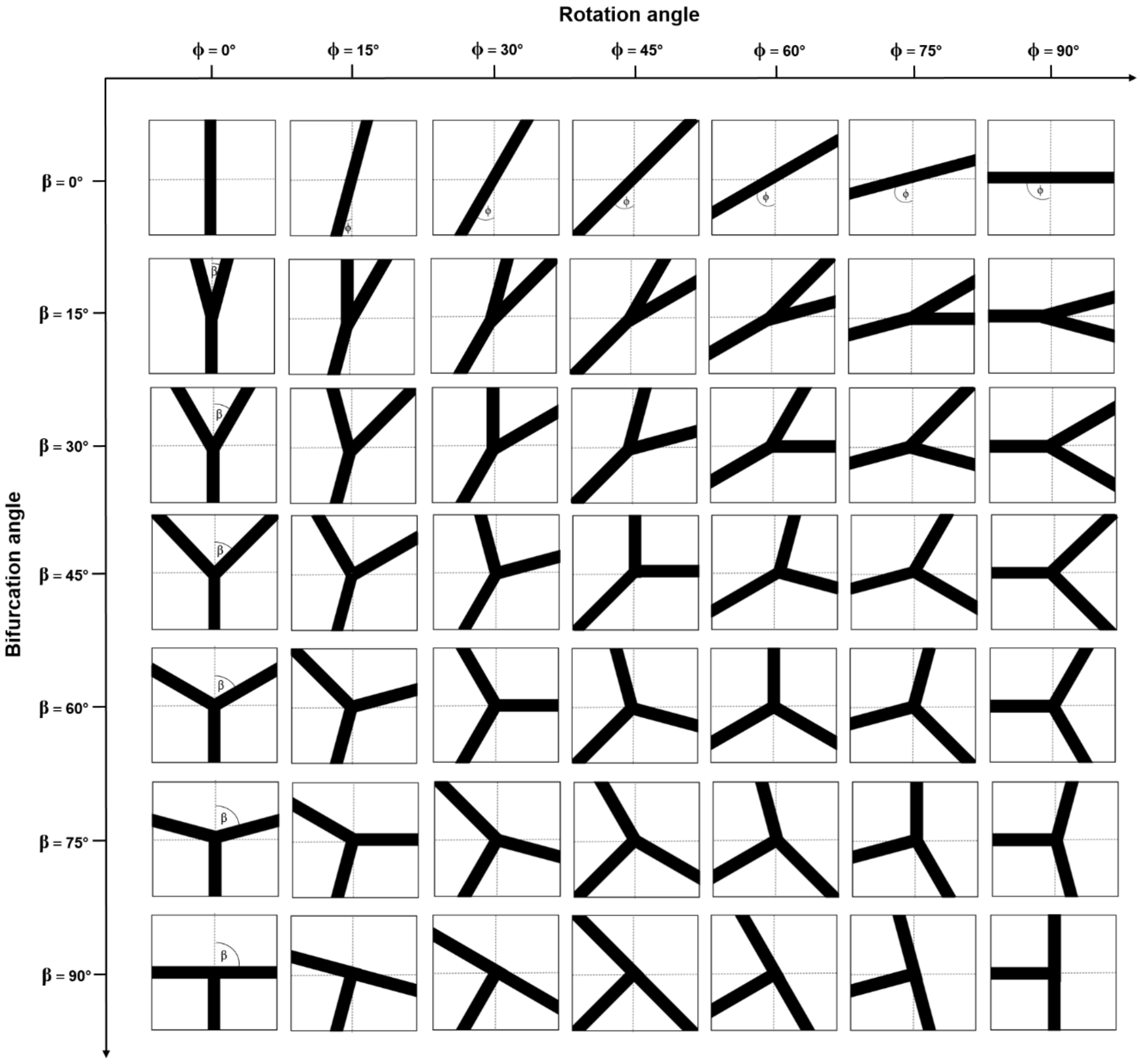

Supplementary Fig. S1. Depiction of the cylinder fork model at different bifurcation and rotation angles. The cylinder fork model is displayed at bifurcation angles β and rotation angles ϕ both varying from 0° to 90°.

Supplementary Figure S2

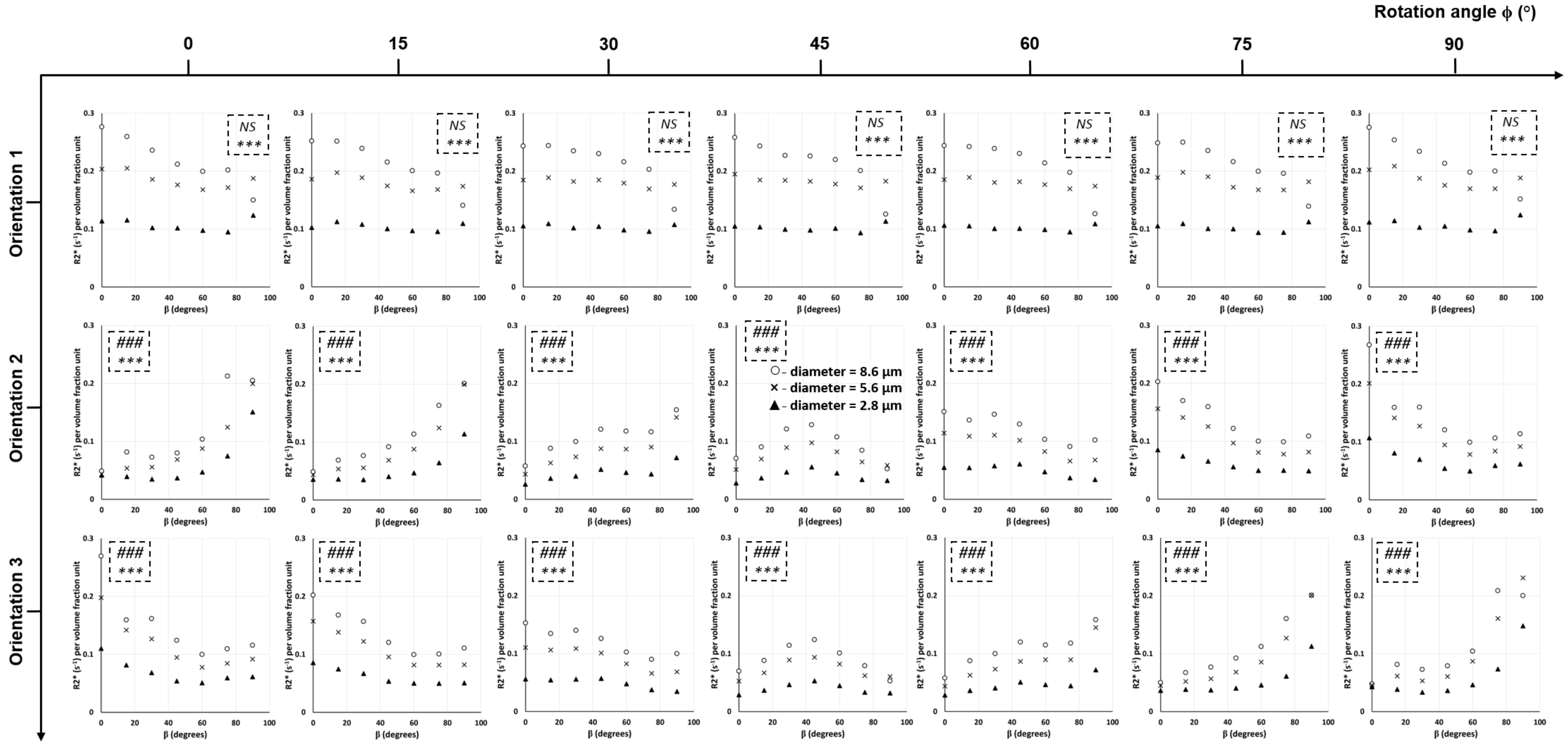

Supplementary Fig. S2. Vessel size effect on R2* relationship with the bifurcation angle. Plots of R2* per volume fraction unit as a function of the bifurcation angle β are shown with three different vessel diameters at three different orientations and rotation angles varying from 0° to 90°. The diffusion rate and susceptibility value are 1×10^{-10} m^2/s and 3×10^{-8} in cgs units, respectively. The statistical significances from a two-way ANOVA are shown for each graph in the

dotted inlet, where the top (#) and bottom (*) symbols show the significant effects of bifurcation angle and vessel diameter on R2*, respectively. ###, *** – $p < 0.001$; NS – not significant. Detailed statistical values are shown in Supplementary Table S8.

Supplementary Figure S3

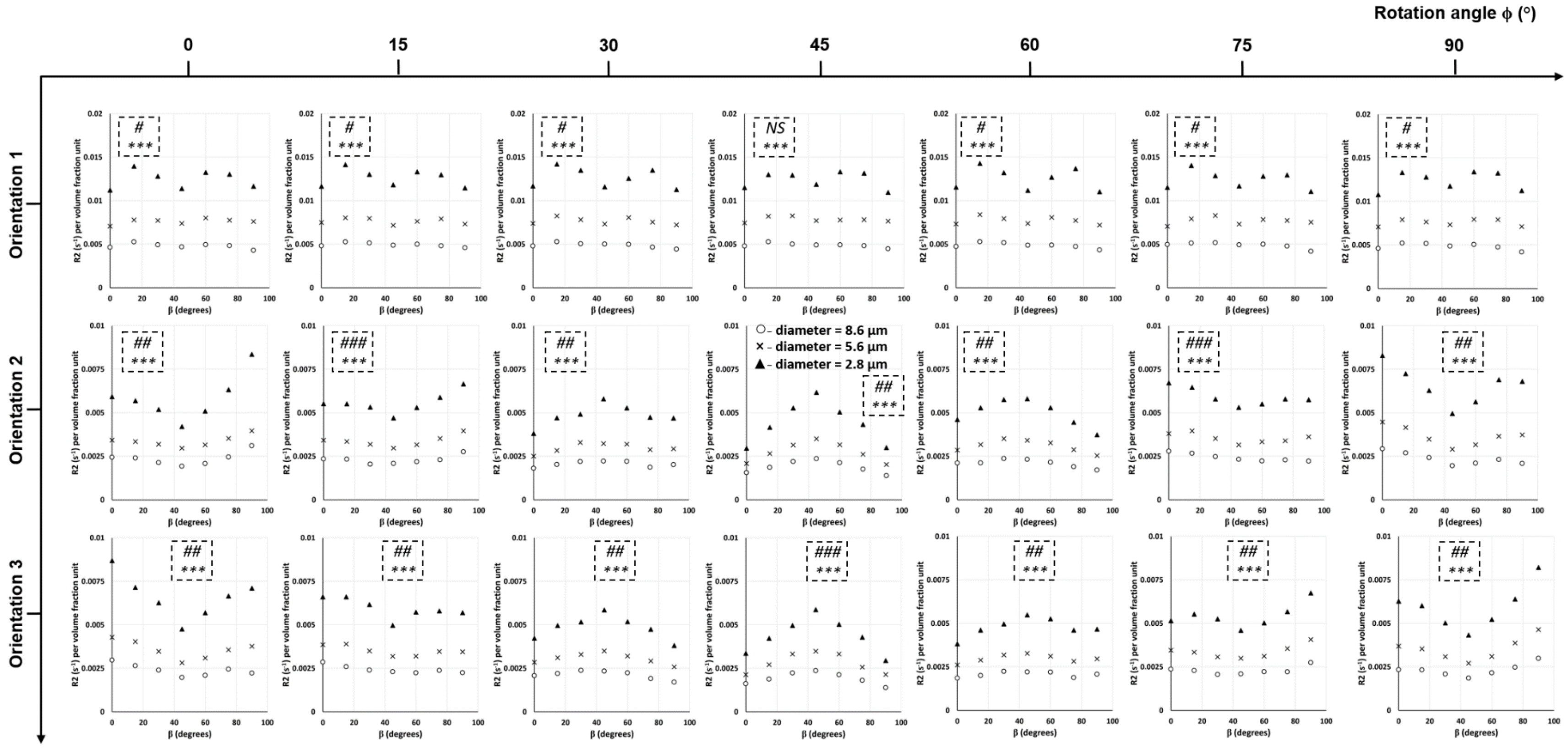

Supplementary Fig. S3. Vessel size effect on R2 relationship with the bifurcation angle. Plots of R2 per volume fraction unit as a function of the bifurcation angle β are shown with three different vessel diameters at three different orientations and rotation angles varying from 0° to 90°. The diffusion rate and susceptibility value are 1×10^{-10} m^2/s and 3×10^{-8} in cgs units, respectively. The statistical significances from a two-way ANOVA are shown for each graph in the

dotted inlet, where the top (#) and bottom (*) symbols show the significant effects of bifurcation angle and vessel diameter on R2, respectively. # – $0.01 < p < 0.05$; ## – $0.001 < p < 0.01$; ###, *** – $p < 0.001$; NS – not significant. Detailed statistical values are shown in Supplementary Table S8.

Supplementary Figure S4

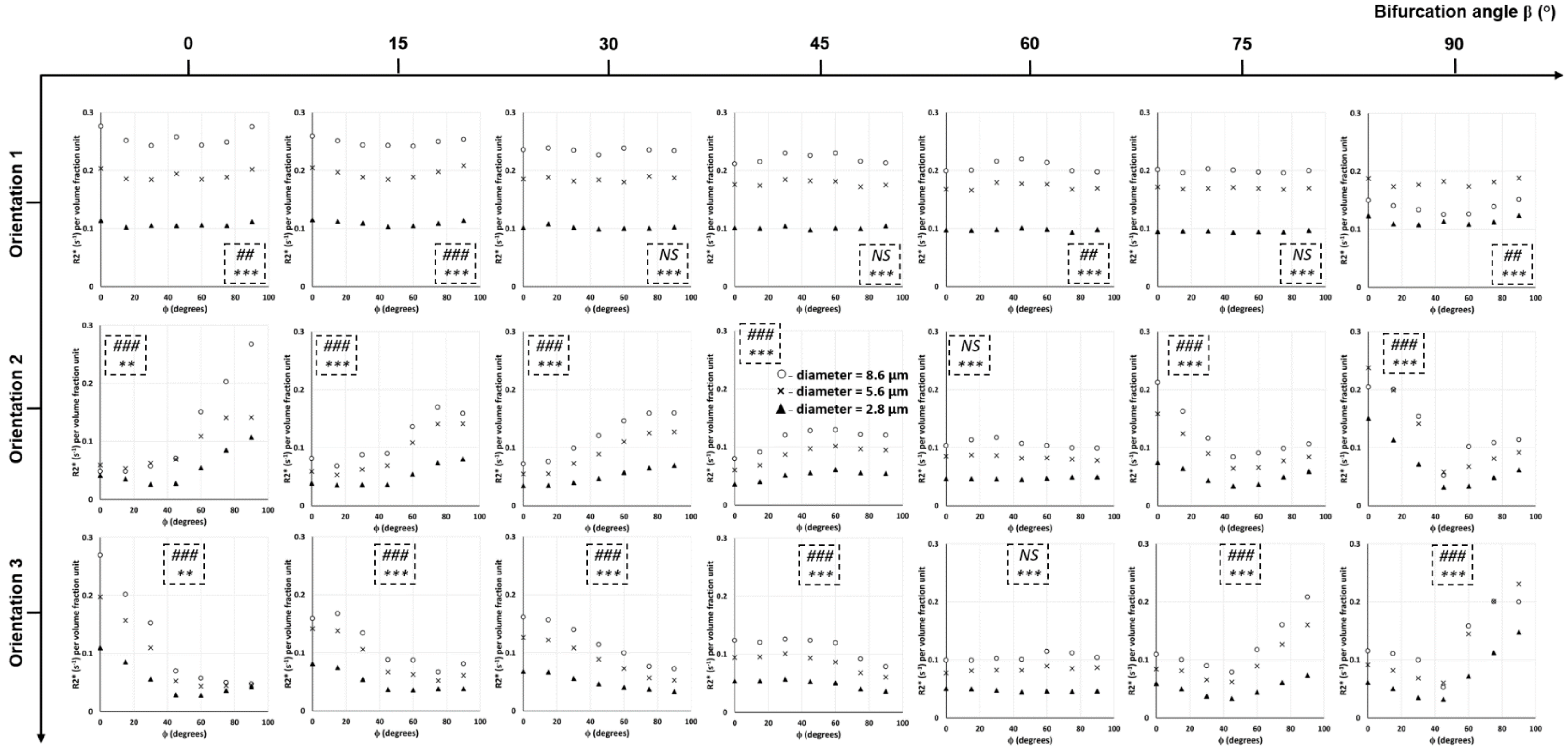

Supplementary Fig. S4. Vessel size effect on R2* relationship with the rotation angle. Plots of R2* per volume fraction unit as a function of the rotation angle ϕ are shown with three different vessel diameters at three different orientations and bifurcation angles varying from 0° to 90°. The diffusion rate and susceptibility value are 1×10^{-10} m^2/s and 3×10^{-8} in cgs units, respectively. The statistical significances from a two-way ANOVA are shown for each graph in the

dotted inlet, where the top (#) and bottom (*) symbols show the significant effects of rotation angle and vessel diameter on R2*, respectively. ##, ** – $0.001 < p < 0.01$; ###, *** – $p < 0.001$; NS – not significant. Detailed statistical values are shown in Supplementary Table S8.

Supplementary Figure S5

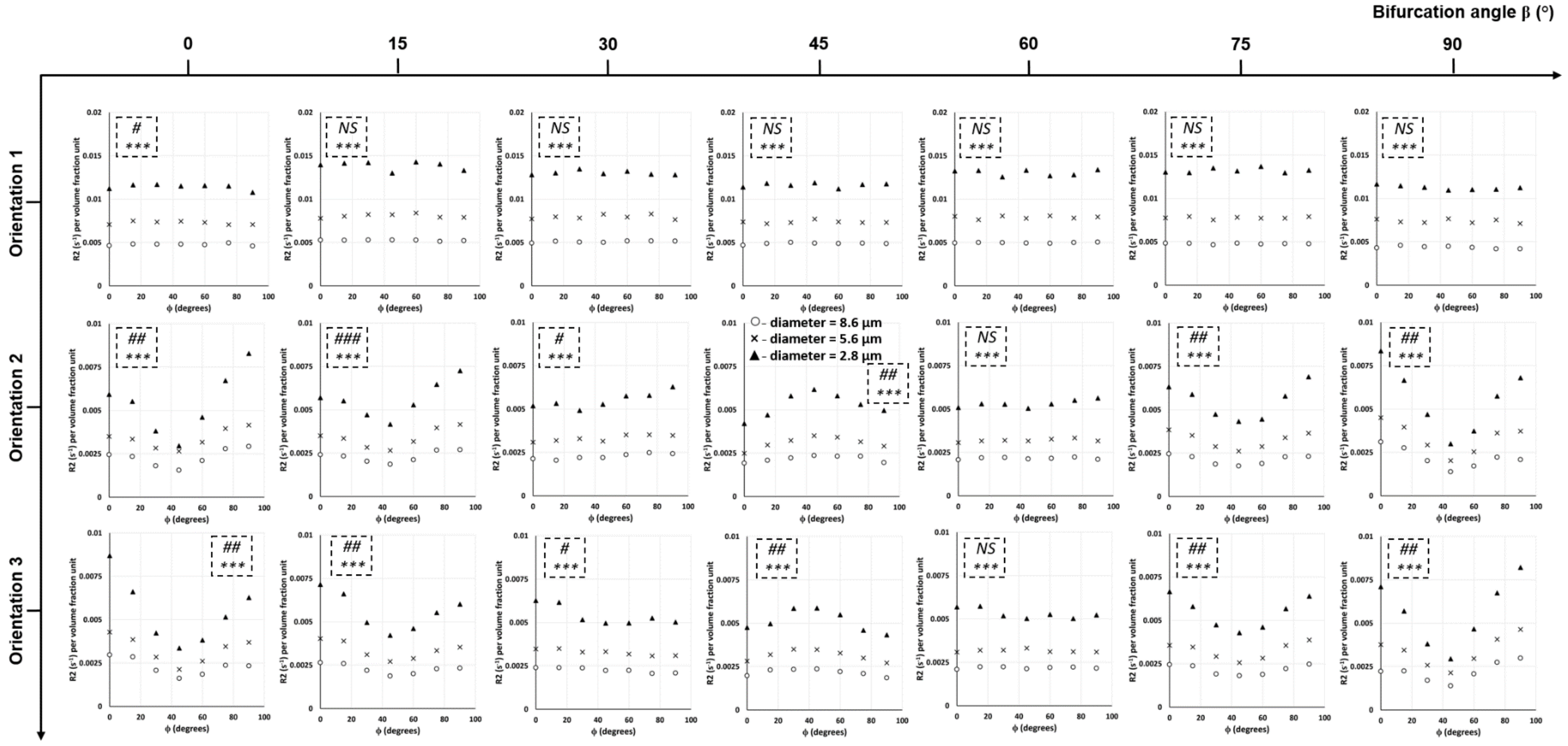

Supplementary Fig. S5. Vessel size effect on R2 relationship with the rotation angle. Plots of R2 per volume fraction unit as a function of the rotation angle ϕ are shown with three different vessel diameters at three different orientations and

bifurcation angles varying from 0° to 90°. The diffusion rate and susceptibility value are 1×10^{-10} m^2/s and 3×10^{-8} in cgs units, respectively. The statistical significances from a two-way ANOVA are shown for each graph in the dotted inlet, where the top (#) and bottom (*) symbols show the significant effects of rotation angle and vessel diameter on R2, respectively. # – $0.01 < p < 0.05$; ## – $0.001 < p < 0.01$; ###, *** – $p < 0.001$; NS – not significant. Detailed statistical values are shown in Supplementary Table S8.

Supplementary Figure S6

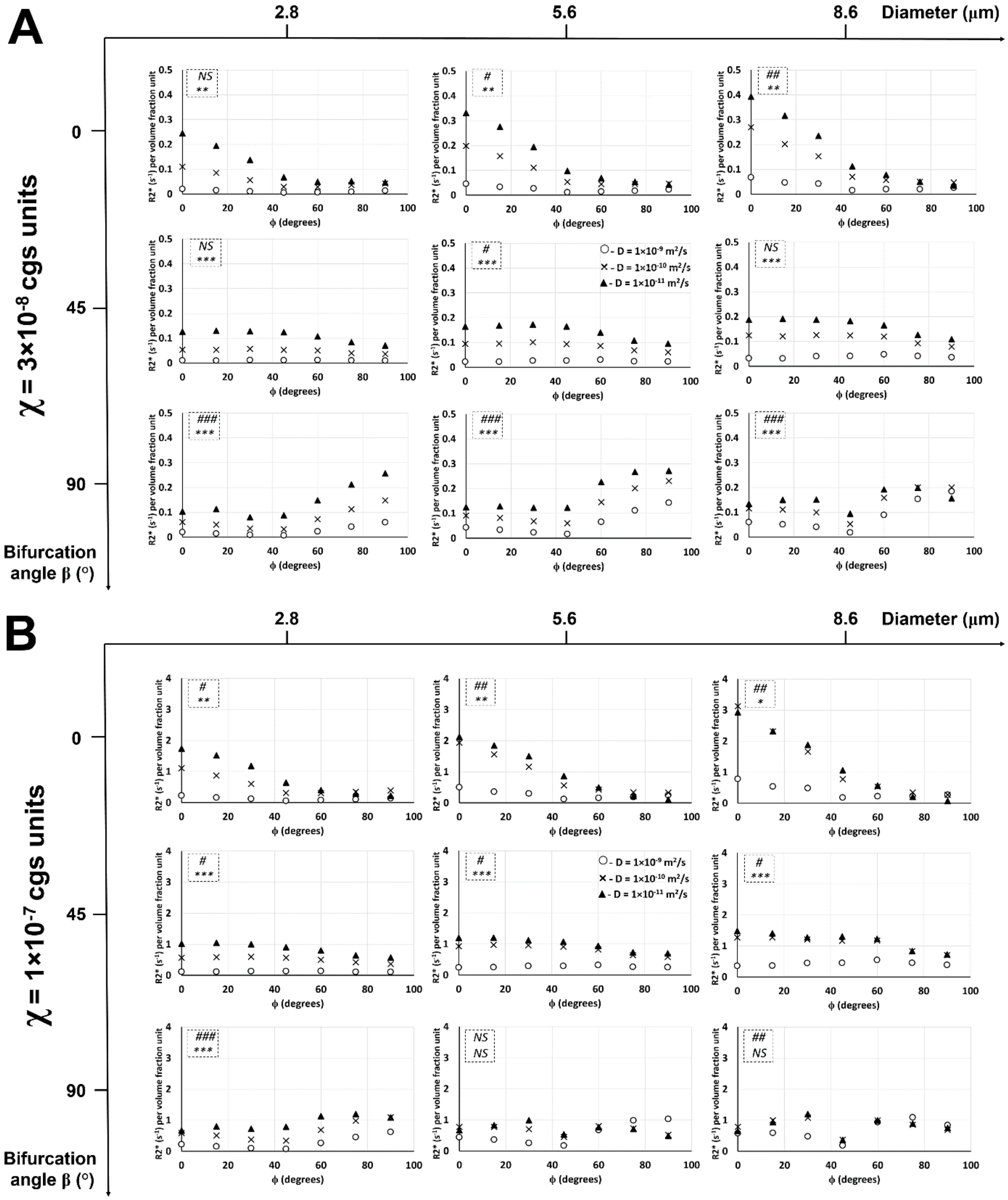

Supplementary Fig. S6. Diffusion rate effect on R2* relationship with the rotation angle in Orientation 3. Plots of <u>R2* per volume fraction unit as a function of the</u>

rotation angle ϕ in Orientation 3 and susceptibility values $\chi = 3\times10^{-8}$ cgs units (A) and $\chi = 1\times10^{-7}$ cgs units (B) are shown with three different diffusion rates at three different bifurcation angles and three different vessel diameters. The statistical significances from a two-way ANOVA are shown for each graph in the dotted inlet, where the top (#) and bottom (*) symbols show the significant effects of rotation angle and diffusion rate on R2*, respectively. # – $0.01 < p < 0.05$; ## – $0.001 < p < 0.01$; *** – $p < 0.001$; NS – not significant. Detailed statistical values are shown in Supplementary Table S9.

Supplementary Figure S7

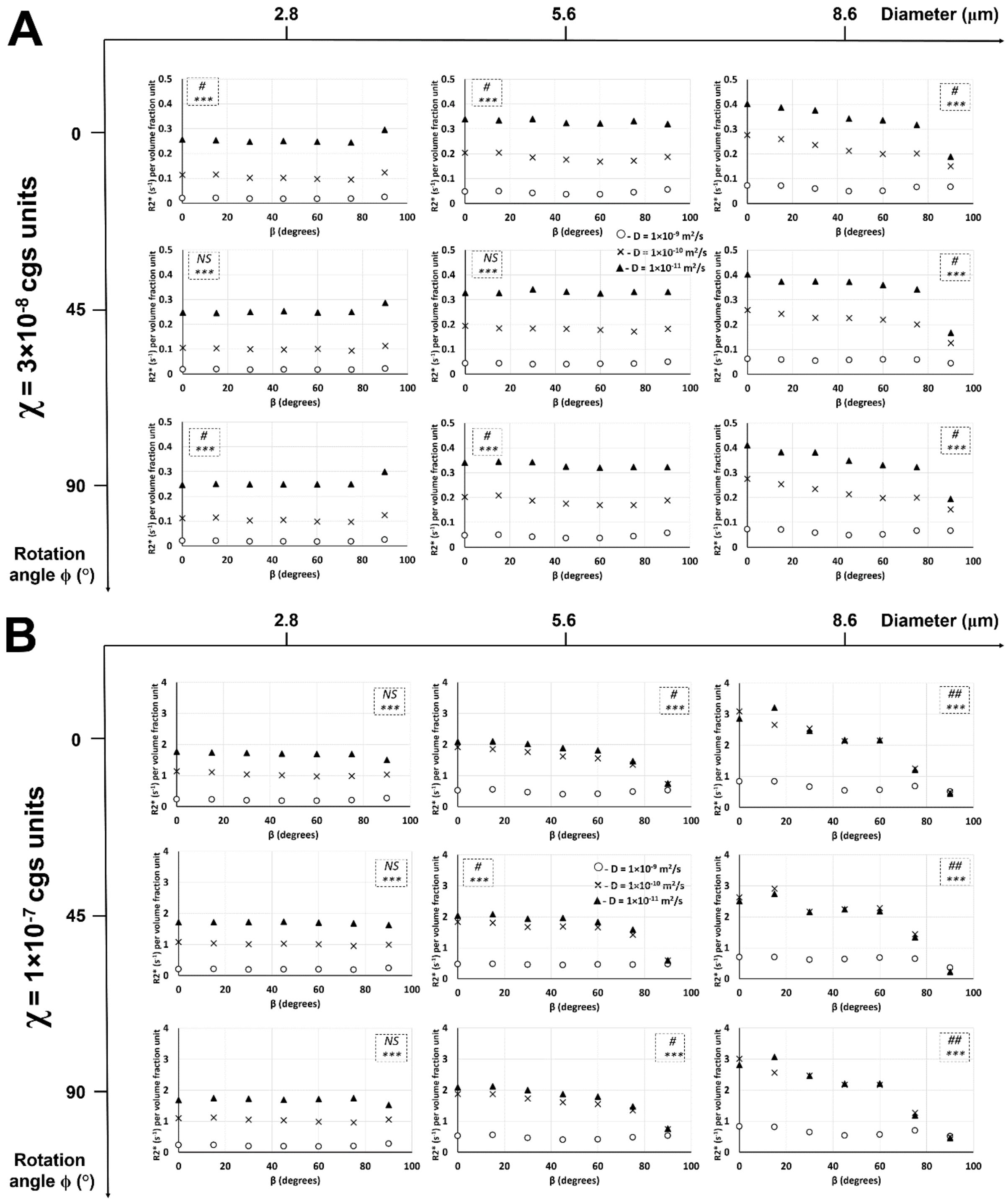

Supplementary Fig. S7. Diffusion rate effect on R2* relationship with the bifurcation angle in Orientation 1. Plots of R2* per volume fraction unit as a function

of the bifurcation angle β in Orientation 1 and susceptibility values $\chi = 3\times10^{-8}$ cgs units (A) and $\chi = 1\times10^{-7}$ cgs units (B) are shown with three different diffusion rates at three different rotation angles and vessel diameters. The statistical significances from a two-way ANOVA are shown for each graph in the dotted inlet, where the top (#) and bottom (*) symbols show the significant effects of bifurcation angle and diffusion rate on R2*, respectively. #, * – $0.01 < p < 0.05$; ##, ** – $0.001 < p < 0.01$; ###, *** – $p < 0.001$; NS – not significant. Detailed statistical values are shown in Supplementary Table S10.

Supplementary Table S1: Summary of the statistical values from a two-way ANOVA performed on Fig. 2 data.

**Figure 2A**			**ϕ = 0°**		**ϕ = 45°**		**ϕ = 90°**	
		df	*F*	*p-value*	*F*	*p-value*	*F*	*p-value*
**Orientation 1**	β	6	1.920	0.1584	1.015	0.4601	1.833	0.1750
	Diameter	2	43.01	3.366E-06	36.33	8.105E-06	43.03	3.359E-06
**Orientation 2**	β	6	18.01	2.336E-05	9.954	4.509E-04	11.90	1.912E-04
	Diameter	2	9.722	3.089E-03	54.65	9.377E-07	26.22	4.169E-05
**Orientation 3**	β	6	12.32	1.613E-04	9.734	5.008E-04	18.00	2.339E-05
	Diameter	2	27.95	3.046E-05	56.61	7.749E-07	10.14	2.637E-03
**Figure 2B**			**ϕ = 0°**		**ϕ = 45°**		**ϕ = 90°**	
		df	*F*	*p-value*	*F*	*p-value*	*F*	*p-value*
**Orientation 1**	β	6	3.465	3.183E-02	2.820	5.973E-02	4.710	1.091E-02
	Diameter	2	446.0	5.472E-12	474.3	3.800E-12	467.5	4.142E-12
**Orientation 2**	β	6	7.650	1.503E-03	7.567	1.576E-03	7.241	1.911E-03
	Diameter	2	92.62	5.072E-08	60.07	5.610E-07	189.3	8.410E-10
**Orientation 3**	β	6	5.570	5.708E-03	9.731	5.017E-04	7.868	1.326E-03
	Diameter	2	130.9	7.091E-09	87.34	7.053E-08	110.5	1.866E-08

Note: *df* = degrees of freedom; *F* = F value. Significant *p*-values are shaded in gray cells. $\alpha = 0.05$ was used as the significance level for all data sets.

Supplementary Table S2: Summary of the statistical values from a two-way ANOVA performed on Fig. 3 data.

**Figure 3A**			**Diameter = 2.8μm**		**Diameter = 5.6μm**		**Diameter = 8.6μm**	
		df	*F*	*p-value*	*F*	*p-value*	*F*	*p-value*
**β = 0°**	ϕ	6	2.739	6.489E-02	10.59	3.365E-04	9.651	5.213E-04
	D	2	8965	8.950E-20	14290	5.477E-21	6193	8.223E-19
**β = 45°**	ϕ	6	0.0476	0.9994	11.73	2.052E-04	12.40	1.563E-04
	D	2	18570	1.136E-21	42630	7.770E-24	9718	5.517E-20
**β = 90°**	ϕ	6	2.349	9.810E-02	1.267	0.3412	29.72	1.575E-06
	D	2	4095	9.803E-18	5135	2.528E-18	2927	7.327E-17
**Figure 3B**			**Diameter = 2.8μm**		**Diameter = 5.6μm**		**Diameter = 8.6μm**	
		df	*F*	*p-value*	*F*	*p-value*	*F*	*p-value*
**β = 0°**	ϕ	6	4.871	9.611E-03	8.806	7.974E-04	6.532	2.970E-03
	D	2	8084	1.664E-19	5618	1.474E-18	440.4	5.896E-12
**β = 45°**	ϕ	6	1.379	0.2987	7.104	2.076E-03	12.90	1.281E-04
	D	2	24080	2.390E-22	13000	9.640E-21	5889	1.112E-18
**β = 90°**	ϕ	6	0.2807	0.9352	12.17	1.714E-04	40.94	2.634E-07
	D	2	1477	4.393E-15	145.1	3.927E-09	21.08	1.183E-04

Note: *df* = degrees of freedom; *F* = F value. Significant *p*-values are shaded in gray cells. $\alpha = 0.05$ was used as the significance level for all data sets.

Supplementary Table S3: Summary of the statistical values from a two-way ANOVA performed on Fig. 4 data.

Figure 4A			Diameter = 2.8μm		Diameter = 5.6μm		Diameter = 8.6μm	
		df	*F*	*p-value*	*F*	*p-value*	*F*	*p-value*
β = 0°	ϕ	6	2.739	6.489E-02	10.59	3.365E-04	9.651	5.213E-04
	D	2	8965	8.950E-20	14290	5.477E-21	6193	8.223E-19
β = 45°	ϕ	6	0.0476	0.9993	11.73	2.052E-04	12.40	1.563E-04
	D	2	18570	1.136E-21	42630	7.770E-24	9718	5.517E-20
β = 90°	ϕ	6	2.349	9.810E-02	1.267	0.3412	29.72	1.575E-06
	D	2	4095	9.803E-18	5135	2.528E-18	2927	7.327E-17
**Figure 4B**			**Diameter = 2.8μm**		**Diameter = 5.6μm**		**Diameter = 8.6μm**	
		df	*F*	*p-value*	*F*	*p-value*	*F*	*p-value*
β = 0°	ϕ	6	4.871	9.611E-03	8.806	7.974E-04	6.532	2.970E-03
	D	2	8084	1.664E-19	5618	1.474E-18	440.4	5.896E-12
β = 45°	ϕ	6	1.379	0.2987	7.104	2.076E-03	12.90	1.281E-04
	D	2	24080	2.390E-22	13000	9.640E-21	5889	1.112E-18
β = 90°	ϕ	6	0.2807	0.9352	12.17	1.714E-04	40.94	2.634E-07
	D	2	1477	4.393E-15	145.1	3.927E-09	21.08	1.183E-04

Note: *df* = degrees of freedom; *F* = F value. Significant *p*-values are shaded in gray cells. α = 0.05 was used as the significance level for all data sets.

Supplementary Table S4: Summary of the statistical values from a two-way ANOVA performed on Fig. 5 data.

Figure 5A			Diameter = 2.8μm		Diameter = 5.6μm		Diameter = 8.6μm	
		df	*F*	*p-value*	*F*	*p-value*	*F*	*p-value*
ϕ = 0°	β	6	2.611	7.410E-02	3.608	2.789E-02	4.313	1.507E-02
	D	2	46.21	2.304E-06	31.01	1.816E-05	27.06	3.574E-05
ϕ = 45°	β	6	4.128	1.760E-02	8.067	1.186E-03	10.20	4.025E-04
	D	2	193.2	7.457E-10	276.5	9.177E-11	156.2	2.558E-09
ϕ = 90°	β	6	6.558	2.921E-03	24.09	4.960E-06	17.92	2.393E-05
	D	2	14.37	6.523E-04	21.45	1.091E-04	8.702	4.621E-03

Figure 5B			Diameter = 2.8μm		Diameter = 5.6μm		Diameter = 8.6μm	
		df	*F*	*p-value*	*F*	*p-value*	*F*	*p-value*
ϕ = 0°	β	6	3.026	4.856E-02	4.575	1.215E-02	5.285	7.024E-03
	D	2	36.94	7.446E-06	21.84	1.003E-04	12.85	1.039E-03
ϕ = 45°	β	6	7.965	1.256E-03	7.201	1.958E-03	8.259	1.067E-03
	D	2	502.0	2.716E-12	122.7	1.026E-08	53.94	1.006E-06
ϕ = 90°	β	6	9.412	5.862E-04	4.102	1.801E-02	9.364	6.003E-04
	D	2	16.31	3.784E-04	0.9861	0.4013	1.466	0.2693

Note: *df* = degrees of freedom; *F* = F value. Significant *p*-values are shaded in gray cells. α = 0.05 was used as the significance level for all data sets.

Supplementary Table S5: Summary of the statistical values from a two-way ANOVA performed on Fig. 6 data.

**Figure 6A**			**Diameter = 2.8μm**		**Diameter = 5.6μm**		**Diameter = 8.6μm**	
		df	*F*	*p-value*	*F*	*p-value*	*F*	*p-value*
**$\chi = 3\times10^{-8}$**	ϕ	6	0.5172	0.7847	0.1730	0.9792	0.7112	0.6477
	D	2	1027	3.845E-14	2527	1.766E-16	2382	2.516E-16
**$\chi = 1\times10^{-7}$**	ϕ	6	0.4745	0.8148	0.3029	0.92358295	0.7589	0.6153
	D	2	1050	3.361E-14	3814	1.501E-17	2319	2.956E-16
**$\chi = 4\times10^{-7}$**	ϕ	6	0.4171	0.8539	0.7667	0.6101	0.8201	0.5752
	D	2	4419	6.212E-18	2598	1.497E-16	1524	3.633E-15
**Figure 6B**			**Diameter = 2.8μm**		**Diameter = 5.6μm**		**Diameter = 8.6μm**	
		df	*F*	*p-value*	*F*	*p-value*	*F*	*p-value*
**$\chi = 3\times10^{-8}$**	β	6	3.102	4.502E-02	0.8243	0.5725	1.305	0.3262
	D	2	357.7	2.016E-11	555.6	1.488E-12	107.8	2.145E-08
**$\chi = 1\times10^{-7}$**	β	6	2.080	0.1321	1.362	0.3047	1.371	0.3014
	D	2	256.0	1.443E-10	550.6	1.569E-12	95.75	4.204E-08
**$\chi = 4\times10^{-7}$**	β	6	1.758	0.1910	1.526	0.2507	1.546	0.2448
	D	2	642.7	6.260E-13	161.3	2.125E-09	68.52	2.725E-07

Note: *df* = degrees of freedom; *F* = F value. Significant *p*-values are shaded in gray cells. $\alpha = 0.05$ was used as the significance level for all data sets.

Supplementary Table S6: Summary of the statistical values from a two-way ANOVA performed on Fig. 7 data.

**Figure 7A**			**Diameter = 2.8μm**		**Diameter = 5.6μm**		**Diameter = 8.6μm**	
		df	*F*	*p-value*	*F*	*p-value*	*F*	*p-value*
**$\chi = 3\times10^{-8}$**	ϕ	6	6.442	3.149E-03	2.725	6.579E-02	2.208	0.1145
	D	2	30.62	1.935E-05	42.24	3.700E-06	56.00	8.210E-07
**$\chi = 1\times10^{-7}$**	ϕ	6	6.620	2.806E-03	2.648	7.132E-02	2.191	0.1167
	D	2	33.44	1.240E-05	42.45	3.607E-06	57.33	7.231E-07
**$\chi = 4\times10^{-7}$**	ϕ	6	4.334	1.480E-02	2.091	0.1305	1.860	0.1696
	D	2	33.15	1.295E-05	44.38	2.855E-06	57.76	6.943E-07
**Figure 7B**			**Diameter = 2.8μm**		**Diameter = 5.6μm**		**Diameter = 8.6μm**	
		df	*F*	*p-value*	*F*	*p-value*	*F*	*p-value*
**$\chi = 3\times10^{-8}$**	ϕ	6	8.156	1.129E-03	3.962	2.033E-02	2.818	5.980E-02
	D	2	341.7	2.640E-11	466.6	4.186E-12	660.3	5.334E-13
**$\chi = 1\times10^{-7}$**	ϕ	6	8.725	8.318E-04	4.159	1.715E-02	2.675	6.934E-02
	D	2	371.5	1.613E-11	474.5	3.791E-12	688.2	4.168E-13
**$\chi = 4\times10^{-7}$**	ϕ	6	8.219	1.091E-03	2.930	5.340E-02	2.181	0.1180
	D	2	457.3	4.717E-12	589.6	1.045E-12	745.4	2.593E-13

Note: *df* = degrees of freedom; *F* = F value. Significant *p*-values are shaded in gray cells. $\alpha = 0.05$ was used as the significance level for all data sets.

Supplementary Table S7: Summary of the statistical values from a two-way ANOVA performed on Fig. 8 data.

Figure 8A			Diameter = 2.8µm		Diameter = 5.6µm		Diameter = 8.6µm	
		df	*F*	*p-value*	*F*	*p-value*	*F*	*p-value*
**χ = 3×10^{-8}**	β	6	6.174	3.758E-03	2.827	5.928E-02	1.978	0.1482
	D	2	108.1	2.115E-08	120.3	1.149E-08	92.51	5.105E-08
**χ = 1×10^{-7}**	β	6	6.475	3.083E-03	2.573	7.714E-02	1.921	0.1581
	D	2	115.5	1.450E-08	103.8	2.669E-08	85.59	7.903E-08
**χ = 4×10^{-7}**	β	6	4.540	1.250E-02	1.998	0.1448	1.658	0.2147
	D	2	124.1	9.601E-09	93.30	4.867E-08	65.74	3.422E-07
**Figure 8B**			**Diameter = 2.8µm**		**Diameter = 5.6µm**		**Diameter = 8.6µm**	
		df	*F*	*p-value*	*F*	*p-value*	*F*	*p-value*
**χ = 3×10^{-8}**	β	6	11.80	1.989E-04	4.252	1.585E-02	2.424	9.044E-02
	D	2	99.67	3.350E-08	93.48	4.812E-08	71.50	2.153E-07
**χ = 1×10^{-7}**	β	6	10.82	3.034E-04	4.266	1.566E-02	2.368	9.608E-02
	D	2	91.65	5.381E-08	91.88	5.307E-08	70.57	2.314E-07
**χ = 4×10^{-7}**	β	6	5.891	4.554E-03	2.516	8.194E-02	2.063	0.1347
	D	2	86.64	7.382E-08	101.1	3.093E-08	85.55	7.924E-08

Note: *df* = degrees of freedom; *F* = F value. Significant *p*-values are shaded in gray cells. α = 0.05 was used as the significance level for all data sets.

Supplementary Table S8: Summary of the statistical values from a two-way ANOVA performed on Supplementary Figs. S2-S5 data.

Figure S2			ϕ = 0°		ϕ = 15°		ϕ = 30°		ϕ = 45°		ϕ = 60°		ϕ = 75°		ϕ = 90°	
		df	*F*	*p-value*	*F*	*p-value*	*F*	*p-value*	*F*	*p-value*	*F*	*p-value*	*F*	*p-value*	*F*	*p-value*
**Orientation 1**	β	6	1.920	0.1584	1.954	0.1523	1.342	0.3123	1.015	0.4601	1.273	0.3389	1.572	0.2375	1.833	0.1750
	Diameter	2	43.01	3.366E-06	50.43	1.445E-06	49.00	1.685E-06	36.33	8.105E-06	40.79	4.449E-06	46.82	2.149E-06	43.03	3.359E-06
**Orientation 2**	β	6	18.01	2.336E-05	20.52	1.173E-05	17.68	2.573E-05	9.954	4.509E-04	13.17	1.156E-04	13.41	1.057E-04	11.90	1.912E-04
	Diameter	2	9.722	3.089E-03	18.59	2.108E-04	67.17	3.040E-07	54.65	9.377E-07	131.5	6.903E-09	55.58	8.556E-07	26.22	4.169E-05
**Orientation 3**	β	6	12.32	1.613E-04	14.26	7.761E-05	12.31	1.615E-04	9.734	5.008E-04	16.08	4.209E-05	18.58	1.980E-05	18.00	2.339E-05
	Diameter	2	27.95	3.046E-05	60.41	5.438E-07	129.0	7.712E-09	56.61	7.749E-07	58.37	6.558E-07	16.90	3.238E-04	10.14	2.637E-03

Figure S3			ϕ = 0°		ϕ = 15°		ϕ = 30°		ϕ = 45°		ϕ = 60°		ϕ = 75°		ϕ = 90°	
		df	*F*	*p-value*	*F*	*p-value*	*F*	*p-value*	*F*	*p-value*	*F*	*p-value*	*F*	*p-value*	*F*	*p-value*
**Orientation 1**	β	6	3.465	3.183E-02	3.483	3.129E-02	3.063	4.678E-02	2.820	5.973E-02	3.596	2.820E-02	3.223	4.005E-02	4.710	1.091E-02
	Diameter	2	446.0	5.472E-12	500.2	2.774E-12	355.5	2.090E-11	474.3	3.800E-12	300.1	5.669E-11	390.5	1.200E-11	467.5	4.142E-12
**Orientation 2**	β	6	7.650	1.503E-03	10.10	4.211E-04	5.187	7.556E-03	7.567	1.576E-03	7.188	1.973E-03	8.780	8.084E-04	7.241	1.911E-03
	Diameter	2	92.62	5.072E-08	452.0	5.053E-12	215.3	3.969E-10	60.07	5.610E-07	186.6	9.128E-10	616.5	8.022E-13	189.3	8.410E-10
**Orientation 3**	β	6	5.570	5.708E-03	6.576	2.888E-03	7.310	1.833E-03	9.731	5.017E-04	5.799	4.855E-03	7.472	1.666E-03	7.868	1.326E-03
	Diameter	2	130.9	7.091E-09	417.7	8.063E-12	206.8	5.022E-10	87.34	7.053E-08	281.8	8.212E-11	258.1	1.374E-10	110.5	1.866E-08

Figure S4			β = 0°		β = 15°		β = 30°		β = 45°		β = 60°		β = 75°		β = 90°	
		df	*F*	*p-value*	*F*	*p-value*	*F*	*p-value*	*F*	*p-value*	*F*	*p-value*	*F*	*p-value*	*F*	*p-value*
**Orientation 1**	ϕ	6	7.226	1.928E-03	13.98	8.585E-05	2.304	0.1030	2.533	8.052E-02	5.133	7.871E-03	2.334	9.973E-02	7.896	1.305E-03
	Diameter	2	1304	9.243E-15	3952	1.213E-17	3686	1.844E-17	1223	1.357E-14	1233	1.292E-14	8042	1.717E-19	410.0	9.008E-12
**Orientation 2**	ϕ	6	12.81	1.328E-04	19.85	1.398E-05	15.38	5.280E-05	20.08	1.315E-05	1.436	0.2789	9.965	4.486E-04	31.28	1.187E-06
	Diameter	2	9.150	3.859E-03	43.00	3.372E-06	57.35	7.219E-07	213.9	4.129E-10	340.5	2.697E-11	31.49	1.681E-05	25.33	4.933E-05
**Orientation 3**	ϕ	6	12.69	1.393E-04	20.10	1.309E-05	15.70	4.759E-05	16.62	3.541E-05	1.378	0.2992	9.895	4.638E-04	29.68	1.585E-06
	Diameter	2	8.826	4.393E-03	41.70	3.962E-06	58.38	6.554E-07	200.0	6.097E-10	342.0	2.629E-11	31.36	1.716E-05	25.65	4.644E-05

Figure S5			β = 0°		β = 15°		β = 30°		β = 45°		β = 60°		β = 75°		β = 90°	
		df	*F*	*p-value*	*F*	*p-value*	*F*	*p-value*	*F*	*p-value*	*F*	*p-value*	*F*	*p-value*	*F*	*p-value*
**Orientation 1**	ϕ	6	3.935	2.081E-02	1.248	0.3491	1.004	0.4659	1.427	0.2820	0.5114	0.7889	0.3701	0.8843	1.229	0.3572
	Diameter	2	3042	5.820E-17	1464	4.618E-15	2551	1.668E-16	2777	1.004E-16	1965	7.958E-16	3061	5.610E-17	1958	8.126E-16
**Orientation 2**	ϕ	6	6.878	2.385E-03	8.510	9.321E-04	4.568	1.222E-02	6.010	4.197E-03	2.205	0.1149	6.284	3.492E-03	7.158	2.010E-03
	Diameter	2	37.88	6.535E-06	139.2	4.975E-09	459.2	4.606E-12	223.6	3.188E-10	1375	6.727E-15	132.8	6.514E-09	40.96	4.352E-06
**Orientation 3**	ϕ	6	5.520	5.917E-03	6.715	2.643E-03	3.060	4.695E-02	7.015	2.192E-03	0.7448	0.6248	7.829	1.356E-03	6.874	2.392E-03
	Diameter	2	31.53	1.669E-05	117.6	1.307E-08	245.4	1.850E-10	278.7	8.767E-11	509.2	2.494E-12	166.6	1.768E-09	39.68	5.137E-06

Note: *df* = degrees of freedom; *F* = F value. Significant *p*-values are shaded in gray cells. α = 0.05 was used as the significance level for all data sets.

Supplementary Table S9: Summary of the statistical values from a two-way ANOVA performed on Supplementary Fig. S6 data.

Figure S6A			Diameter = 2.8µm		Diameter = 5.6µm		Diameter = 8.6µm	
		df	*F*	*p-value*	*F*	*p-value*	*F*	*p-value*
β = 0°	ϕ	6	2.928	5.353E-02	4.301	1.522E-02	5.286	7.018E-03
	D	2	11.95	1.393E-03	10.19	2.591E-03	9.124	3.899E-03
β = 45°	ϕ	6	2.331	9.997E-02	3.081	4.599E-02	2.962	5.174E-02
	D	2	119.0	1.225E-08	103.0	2.782E-08	93.19	4.899E-08
β = 90°	ϕ	6	9.249	6.358E-04	41.17	2.554E-07	10.80	3.052E-04
	D	2	41.50	4.061E-06	88.76	6.444E-08	13.87	7.578E-04
**Figure S6B**			**Diameter = 2.8µm**		**Diameter = 5.6µm**		**Diameter = 8.6µm**	
		df	*F*	*p-value*	*F*	*p-value*	*F*	*p-value*
β = 0°	ϕ	6	3.611	2.782E-02	5.901	4.524E-03	7.009	2.200E-03
	D	2	10.95	1.970E-03	8.266	5.536E-03	6.885	1.020E-02
β = 45°	ϕ	6	3.047	4.752E-02	3.761	2.428E-02	3.370	3.478E-02
	D	2	107.8	2.153E-08	82.38	9.792E-08	45.64	2.460E-06
β = 90°	ϕ	6	14.39	7.420E-05	0.9272	0.5094	5.188	7.547E-03
	D	2	70.00	2.422E-07	0.7743	0.4827	1.766	0.2127

Note: *df* = degrees of freedom; *F* = F value. Significant *p*-values are shaded in gray cells. α = 0.05 was used as the significance level for all data sets.

Supplementary Table S10: Summary of the statistical values from a two-way ANOVA performed on Supplementary Fig. S7 data.

Figure S7A			Diameter = 2.8µm		Diameter = 5.6µm		Diameter = 8.6µm	
		df	*F*	*p-value*	*F*	*p-value*	*F*	*p-value*
ϕ = 0°	β	6	4.138	1.746E-02	3.054	4.721E-02	3.264	3.847E-02
	D	2	1407	5.857E-15	2148	4.675E-16	99.73	3.340E-08
ϕ = 45°	β	6	2.825	5.940E-02	0.9086	0.5204	3.863	2.217E-02
	D	2	1959	8.115E-16	4465	5.840E-18	102.6	2.837E-08
ϕ = 90°	β	6	3.469	3.172E-02	4.664	1.132E-02	3.228	3.984E-02
	D	2	1158	1.872E-14	2319	2.955E-16	103.5	2.707E-08
**Figure S7B**			**Diameter = 2.8µm**		**Diameter = 5.6µm**		**Diameter = 8.6µm**	
		df	*F*	*p-value*	*F*	*p-value*	*F*	*p-value*
ϕ = 0°	β	6	1.409	0.2883	3.641	2.706E-02	5.108	8.017E-03
	D	2	1025	3.883E-14	44.41	2.844E-06	18.72	2.047E-04
ϕ = 45°	β	6	1.427	0.2820	3.795	2.354E-02	5.805	4.836E-03
	D	2	4468	5.817E-18	40.62	4.542E-06	20.75	1.272E-04
ϕ = 90°	β	6	0.6429	0.6953	3.658	2.665E-02	4.937	9.137E-03
	D	2	991.6	4.733E-14	45.44	2.519E-06	19.16	1.840E-04

Note: *df* = degrees of freedom; *F* = F value. Significant *p*-values are shaded in gray cells. α = 0.05 was used as the significance level for all data sets.

References

1 Duvernoy, H. M., Delon, S. & Vannson, J. L. Cortical blood vessels of the human brain. *Brain Res Bull* 7, 519-579 (1981).

2 Less, J. R., Skalak, T. C., Sevick, E. M. & Jain, R. K. Microvascular architecture in a mammary carcinoma: branching patterns and vessel dimensions. *Cancer Res* 51, 265-273 (1991).

3 Cassot, F., Lauwers, F., Fouard, C., Prohaska, S. & Lauwers-Cances, V. A novel three-dimensional computer-assisted method for a quantitative study of microvascular networks of the human cerebral cortex. *Microcirculation* **13**, 1-18 (2006).
